# NPKGRIDS: a global georeferenced dataset of N, P_2_O_5_, and K_2_O fertilizer application rates for 173 crops

**DOI:** 10.1038/s41597-024-04030-4

**Published:** 2024-10-30

**Authors:** Thu Ha Nguyen, Fiona H. M. Tang, Giulia Conchedda, Leon Casse, Griffiths Obli-Laryea, Francesco N. Tubiello, Federico Maggi

**Affiliations:** 1 Environmental Engineering, School of Civil Engineering, The University of Sydney, Sydney, New South Wales Australia; 2 Alluvium Consulting, Sydney, New South Wales Australia; 3 School of Environmental and Rural Science, University of New England, Armidale, New South Wales 2351 Australia; 4 Department of Civil Engineering, Monash University, Clayton, 3800 Victoria Australia; 5 Land and Water Division, Food and Agriculture Organization of the United Nations, Viale delle Terme di Caracalla, Rome, 00153 Italy; 6 Statistics Division, Food and Agriculture Organization of the United Nations, Viale delle Terme di Caracalla, Rome, 00153 Italy; 7 Sydney Institute of Agriculture, The University of Sydney, Sydney, NSW 2006 Australia

**Keywords:** Environmental impact, Sustainability

## Abstract

We introduce NPKGRIDS, a new geospatial dataset, providing for the first time data on application rates for all three main plant nutrients, nitrogen (N), phosphorus (P, in terms of phosphorus pentoxide, P_2_O_5_) and potassium (K, in terms of potassium oxide, K_2_O) across 173 crops as of 2020, with a geospatial resolution of 0.05° (approximately 5.6 km at the equator). Development of NPKGRIDS adopted a data fusion approach to integrate crop mask information with eight published datasets of fertilizer application rates, compiled from either georeferenced data or national and subnational statistics. Furthermore, the total applied mass of N, P_2_O_5_, and K_2_O were benchmarked against the country level information from FAO and the International Fertilizers Association (IFA) and validated against data available from National Statistical Offices (NSOs). NPKGRIDS can be used in global modelling, and decision and policy making to help maximize crop yields while reducing environmental impacts.

## Background & Summary

The use of chemical and mineral fertilizers has grown nearly 10-fold over the last sixty years^1^, contributing decisively to the increase in crop and livestock production over the same period, driven by growing global food and feed demand of the expanding world economy^2^. At the same time, over- and non-optimal use of fertilizers has created, by means of spill-over flows from agricultural fields, serious environmental problems potentially affecting the health of ecosystems and people at all scales, from local soil and water pollution, to regional eutrophication hotspots, to marine dead zones at the confluence of major rivers draining important agricultural areas^3–6^. The dual goal of ensuring food supply to meet global demand while reverting and reducing environmental damage is a major challenge for humanity and the planet, one that is foundational to the 2030 Sustainable Development Agenda^6,7^ and the Global Biodiversity Framework^8^, specifically in relation to the need for efficient use of fertilizers to achieve productive and sustainable agriculture.

Two global datasets, FAO^1,9,10^ and IFA^11^, currently provide rich information on nitrogen (N), phosphorus (P), and potassium (K) applications for agriculture, with country-level statistics for the 1961–2022 period, with annual updates. More limited data on crop-specific application rates is also available^12^. This information is a recognized global reference, facilitating analyses of fertilizers use in agriculture and its trends at country, regional and global scale, as demonstrated by dozens of published papers^13^, international reports^14^, sustainability indices^15^ and planetary boundary science^16^.

At the same time, studies concerned with local or regional issues may often require more detailed, subnational scale information, to assess the interactions of fertilizer use with critical co-variants such as, for instance, climatic conditions, soil properties and water flows, ecosystems and crops distribution, farm management typology, infrastructure and population data. In order to address these needs, global spatial fertilizer maps have begun to emerge in the literature^17–24^, largely in the context of informing models of global biogeochemical studies and earth systems science. These products are useful steps in refining information from national to sub-national and grid-level data, though they suffer from a number of important limitations. One is these new maps were typically produced by spatializing already existing national-level information, without incorporating more detailed published data and subnational information from national statistical offices. Another is that the production of such maps requires significant amount of data and computing resources for both development and validation, so that the existing products have largely been one-off efforts, lacking the required coordination needed to facilitate continuous improvement and updates. Indeed, the most widely used geospatial dataset to date, providing application rates of N, P, and K by crop species^25^ (hereby referred to as MFM and standing for Mueller’s *et al*. Fertilizers Maps), is limited to data for the year 2003. Significant changes in agricultural land and fertilizer use in the last 20 years^9^, coupled with momentous changes in computing power and storage space suggest that the times are now mature for implementing a major update of the currently available products.

Here we present the results of a major new effort in data fusion to produce NPKGRIDS, an updated dataset of global gridded application rates of inorganic fertilizers by main plant nutrients: nitrogen (N), phosphorous (P_2_O_5_), and potassium (K_2_O), by crop species, for the year 2020. NPKGRIDS includes the fertilizer application rates of 173 crops at a global spatial resolution of 0.05° (approximately 5.6 km at the equator). The development of NPKGRIDS adopted a data fusion approach to integrate crop mask information recently made available in CROPGRIDS^26,27^ with other relevant published data sources, as follows. First, we searched and collected the available peer-reviewed and national dispatches of crop-specific fertilizer use data, selecting eight datasets with information specifying individual crops or aggregated crop groups in either georeferenced or tabulated formats. We then selected the best-fit dataset for each crop and subnational unit, using the same data fusion optimization process and quality scoring system of CROPGRIDS. Published national statistics of total applied mass from FAO and IFA and national statistical offices were used subsequently for benchmarking NPKGRIDS.

## Methods

**Fig. 1:**
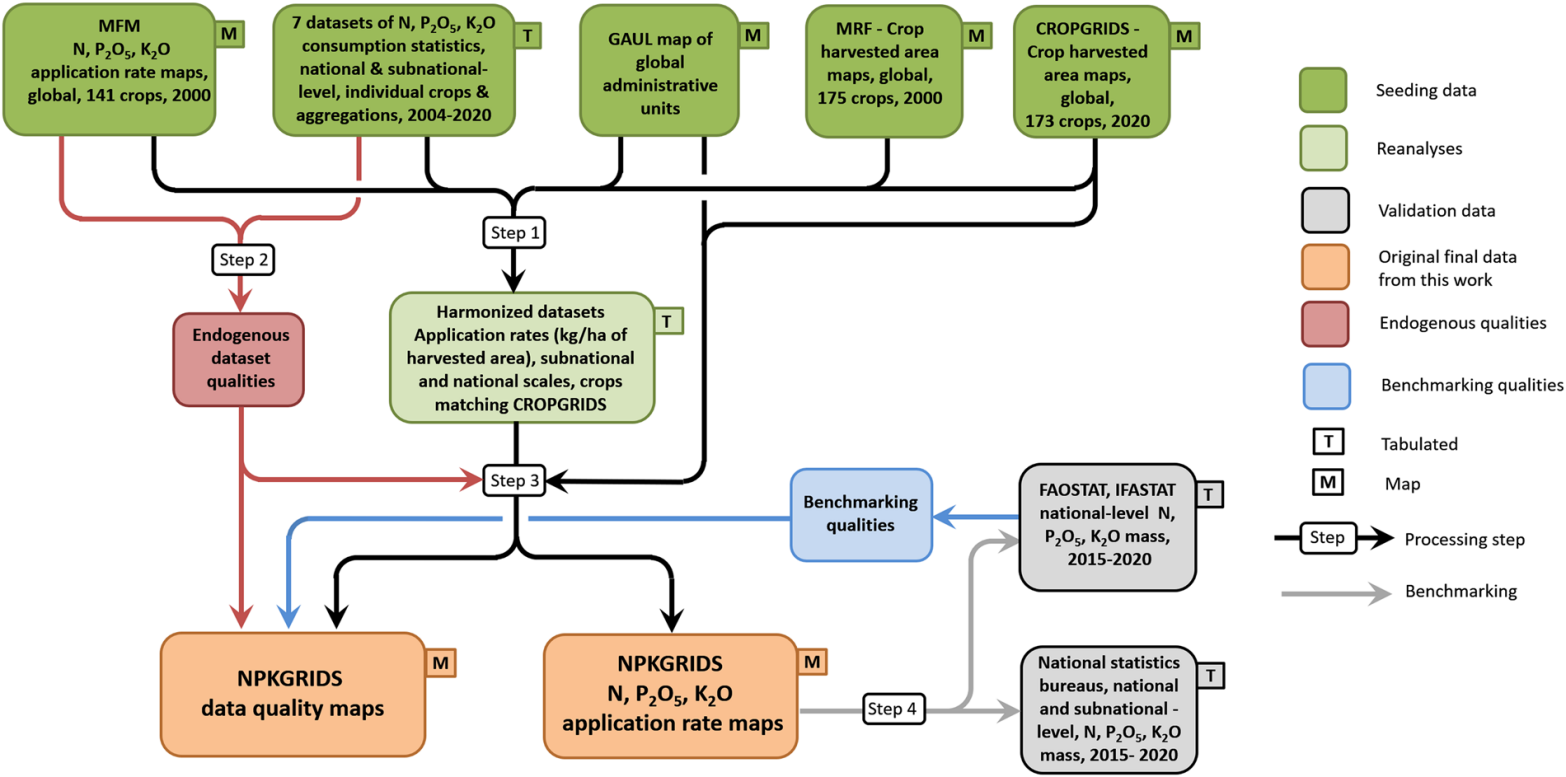
Workflow of the development of NPKGRIDS. Step 1: Harmonization of input datasets into tabular format; Step 2: Determination of endogenous data quality indicators; Step 3: Global spatialization of fertilizer application rates; and Step 4: Validation. MFM: Mueller’s *et al*.^25^ Fertilizers Maps. MRF: Monfreda *et al*.^34^ dataset. GAUL: Global Administrative Unit Layers dataset.

We surveyed and collected georeferenced and tabulated datasets reporting the application rates or applied amounts of N, P and K fertilizers to individual crops at national and/or subnational levels. We only collected datasets from peer-reviewed and national sources for data reliability. We next elaborated these datasets following the workflow depicted in Fig. 1, which includes three main steps: Step 1) harmonization of input datasets into tabular format at the level of subnational units; Step 2) determination of endogenous data quality indicators; and Step 3) global spatialization of fertilizer application rates.

### Input and corollary data sources

The starting point for NPKGRIDS was CROPGRIDS^26,27^, a recently developed georeferenced dataset of crop maps detailing crop location and harvested area. We then researched the available peer-reviewed literature and official national statistics for georeferenced and tabulated fertilizer use datasets specifying individual crops and/or aggregated crop groups. We included only datasets with data vintage more recent than 2003, that is, the latest temporal coverage of MFM^25^, and with crop names matching the FAO Indicative Crop Classification (ICC)^28^. We excluded datasets that were non-crop-specific or containing aggregated crops but without further specification of component crops. The collected datasets provided the mass and/or application rates of total nitrogen (N), total phosphorus (P or P_2_O_5_), and total potassium (K or K_2_O) derived from straight and/or compound fertilizer products. Out of these, we selected eight datasets for N and seven for P_2_O_5_ and K_2_O (Table 1 and Supplementary Table 1). Amongst the selected datasets, the periodic Fertilizer Use By Crop (FUBC)^29^ includes crop-specific and aggregated crop groups across the period 2016–2018 for 63 countries. We separated it into two datasets, one listing only individual (IDV) crops (FUBC18-IDV) and the other listing only aggregated (AGG) crop groups (FUBC18-AGG). The Historic Fertilizer Use By Crop (HFUBC) dataset^12^ combines all fertilizer use by crop data for individual crops and crop groups from IFA and FAO from 1978 to 2018 for 111 countries. Only 12 individual crops in 65 countries from 2006 to 2018 from HFUBC were used in this work. Note that the FUBC18 and HFUBC are national-resolution datasets. Four National Statistical Offices (NSOs) datasets for the United States of America^30^ (US), Belarus^31^ (BY), the United Kingdom^32^ (UK), and Australia^33^ (AU) providing subnational-resolution crop-specific fertilizer data were also included. These eight datasets were used as inputs to construct NPKGRIDS (Table 1).

**Table 1:** List of input used to construct NPKGRIDS.

	Name/Acronym	Description	Use	Reference
1	MFM	Global gridded N, P_2_O_5_, K_2_O application rates [kg/ha of crop harvested area] for 141 crops and pastures at a resolution of 0.0833 degree (~10 km at the equator); reference year 2000.	Input	^25,48^
		Briefly, data was estimated based on national and subnational statistics collected from various international organisations and NSOs, and spatialized based on MRF crop masks. Data granularity at subnational and national levels, i.e., pixel values are uniform in a whole subnational or national unit. Data granularity in a country varies among crops and nutrients. Data quality varies greatly across geographical areas, crop types, and nutrients, depending on the availability of input data.		
		Available at: https://zenodo.org/record/5260732#.YqlfS3ZBy5d		
2	FUBC18-IDV	Tabulated N, P_2_O_5_, K_2_O mass [tonnes], application rates [kg/ha of crop area], crop area [ha] for 43 individual (IDV) crops in 63 countries from 2016 to 2018 at national-level data resolution.	Input	^29^
		Available at: https://www.ifastat.org/consumption/fertilizer-use-by-crop		
3	FUBC18-AGG	Tabulated N, P_2_O_5_, K_2_O mass [tonnes], application rates [kg/ha of crop area], crop area [ha] for 12 clearly defined, aggregated (AGG) crop groups in 39 countries from 2016 to 2018 at national-level data resolution.	Input	^29^
		Available at: https://www.ifastat.org/consumption/fertilizer-use-by-crop		
4	HFUBC	Tabulated N, P_2_O_5_, K_2_O mass [tonnes] and crop area [ha] for 102 crops and crop groups aggregations in 111 countries from 1978 to 2018 at national-level resolution. Only 12 crops in 65 countries from 2006 to 2018 were used in this work.	Input	^12^
		Available at: https://www.ifastat.org/consumption/fertilizer-use-by-crop		
5	US	Tabulated N, P_2_O_5_, K_2_O application rates [pounds/acre], fertilized area [% of harvested area] for 4 crops in the USA from 1964 to 2018 at subnational-level data resolution. Only data from 2007 to 2018 are considered.	Input	^30^
		Available at: https://www.ers.usda.gov/data-products/fertilizer-use-and-price/		
6	AU	Tabulated N applied mass [tonnes] and fertilized area [ha] for 3 crops in Australia in 2016 at subnational-level data resolution.	Input	^33^
		Available at: https://www.abs.gov.au/statistics/industry/agriculture/land-management-and-farming-australia/2016-17#data-download		
7	BY	Tabulated N, P, K applied mass [thousand tonnes] for 3 crops and 5 crop groups in Belarus in 2020 at subnational-level data resolution. Only 3 crops are considered.	Input	^31^
		Available at: http://dataportal.belstat.gov.by/Indicators/Preview?key=140816#		
8	UK	Tabulated N, P_2_O_5_, K_2_O application rate [kg/ha of crop area] for 11 crops and several crop groups in the UK in 2020 at subnational-level data resolution. Only 11 crops and 1 crop group are considered.	Input	^32^
		Available at: https://www.gov.uk/government/statistical-data-sets/british-survey-of-fertiliser-practice-dataset		

Acronyms are: FUBC, Fertilizers Use By Crop; HFUBC, Historic Fertilizer Use by Crop.

Additional, corollary datasets were also used, namely to assist with calculations and spatialization of NPKGRIDS. Specifically, two georeferenced datasets of global crop maps were used to inform crop location and harvested area, i.e., the CROPGRIDS^26,27^ dataset providing maps for 173 crops at 0.05° in 2020 and the Monfreda *et al*.^34^ dataset (hereafter called MRF from the initials of the original authors) providing maps for 175 crops at 0.0833° circa 2000, both using FAO crop species nomenclature. Whenever the selected datasets did not provide fertilizer application rate but only total applied mass, we used national-level crop harvested area publicly available from either NSOs, i.e., CROP-AU^35^ and CROP-BY^36^, or FAOSTAT^37^, to estimate fertilizer application rates as mass per unit of crop harvested area. We used the FAO Global Administrative Unit Layers (GAUL) dataset to identify country and regional (subnational unit) boundaries^38^ (Table 2).

**Table 2:** List of corollary datasets used to construct and benchmark NPKGRIDS.

	Name/Acronym	Description	Use	Reference
9	CROPGRIDS	Global gridded crop harvested area [ha of crop area] for 173 crops at a resolution of 0.05 degree (~5.6 km at the equator); reference year 2020.	Corollary	^26,27^
		Available at: https://figshare.com/articles/dataset/CROPGRIDS/22491997		
10	MRF	Global gridded crop harvested area [ha of crop area] for 175 crops at a resolution of 0.0833 degree (~10 km at the equator); reference year 2000.	Corollary	^34^
		Available at: https://sedac.ciesin.columbia.edu/data/set/aglands-croplands-2000		
11	CROP-AU	Tabulated crop harvested area [ha of crop area] at national level obtained from National Statistics Offices (NSOs) in Australia in 2016.	Corollary	^35^
		Available at: https://www.abs.gov.au/statistics/industry/agriculture/land-management-and-farming-australia/latest-release#data-downloads		
12	CROP-BY	Tabulated crop harvested area [ha of crop area] at national level obtained from National Statistics Offices (NSOs) in Belarus 2020.	Corollary	^36^
		Available at: https://www.belstat.gov.by/en/ofitsialnaya-statistika/real-sector-of-the-economy/selskoe-hozyaistvo/anual_data/sown-area-under-main-agricultural-crops-by-region/		
13	FAOSTAT (crop harvested area)	Tabulated crop harvested area [ha of crop area] at national level obtained from FAOSTAT, from 2006 to 2020.	Corollary	^37^
		Available at: https://www.fao.org/faostat/en/#data/QCL		
13	GAUL	Gridded administrative units for all countries; reference year 2015; level 0 (national level), level 1 (subnational level).	Corollary	^38^
		Available at: https://developers.google.com/earth-engine/datasets/catalog/FAO_GAUL_2015_level1#description		
14	FAOSTAT (N, P, K applied mass)	Tabulated total applied mass (non-crop-specific) of N, P_2_O_5_, K_2_O [tonnes] at national-level for 161 countries, from 2015–2020.	Benchmarking	^41^
		Available at: https://www.fao.org/faostat/en/#data/RFN		
15	IFASTAT (N, P, K applied mass)	Tabulated total applied mass (non-crop-specific) of N, P_2_O_5_, K_2_O [tonnes] at national-level for 110 countries, from 2015–2020.	Benchmarking	^11^
		Available at: https://www.ifastat.org/databases/plant-nutrition		
16	NSO (N, P, K applied mass)	Tabulated total applied mass (non-crop-specific) of N, P_2_O_5_ (or P), K_2_O (or K) [tonnes] at national and subnational levels for 37 countries and 166 subnational units, from 2006–2020, including 32 countries in Europe, India, Pakistan, China, Iran, and Sri Lanka.	Benchmarking	^42–47^
				See details in Supplementary Table 2

Acronyms are: GAUL, Global Administrative Unit Layers; NSO, National Statistical Offices.

### Data harmonization (Step 1)

The eight input datasets (Table 1) were harmonized to a common tabular format for N, P_2_O_5_, and K_2_O application rates in each crop expressed as mass applied per unit crop harvested area. The tabular resolution is at the finest scale of each dataset, i.e., subnational (level 1) for MFM, US, BY, AU, UK and national (level 0) for FUBC18-IDV, FUBC18-AGG, and HFUBC.

For the georeferenced MFM dataset, we first tabulated the application rates using the GAUL level 1 mask at the dataset original resolution, i.e., 0.0833° (~10 km at the equator). In subnational units with missing data of fertilizer application rates, we gap-filled the missing information using the national weighted average application rate *F*_MFM_ [kg ha^−1^] for fertilizer *n*, crop *i* and country *j* calculated as:

1 $${F}_{{\rm{MFM}}}(n,i,j)=\frac{{\sum }_{r{\in }j}[\,{f}_{{\rm{MFM}}}(n,i,j,r)\cdot {A}_{{\rm{MRF}}}(i,j,r)\,]}{{\sum }_{r{\in }j}{A}_{{\rm{MRF}}}(i,j,r)}$$

where *f*_MFM_(*n*,*i*,*j*,*r*) in [kg ha^−1^] is the available application rate of fertilizer *n* for crop *i* in subnational unit *r* of country *j* in the MFM dataset, and *A*_MRF_ is the corresponding harvested area obtained from the MRF dataset.

For all other tabular datasets, the harmonization process consisted in converting the variables to application rates expressed as mass of applied N, P_2_O_5_, and K_2_O per unit crop harvested area. In cases where the datasets only provided information on applied mass, we calculated the application rate using crop-specific harvested areas of the corresponding year sourced from the relevant corollary datasets (Table 2). Specifically, crop harvested area from FAOSTAT was used for HFUBC, FUBC18-IDV and FUBC18-AGG while CROP-AU and CROP-BY were used for the AU and BY datasets, respectively. For the US dataset, which provided information on application rates per unit fertilized area and the percent harvested area being fertilized, we calculated the application rates for the entire harvested area of each crop by multiplication. For the BY dataset, P and K mass were multiplied by 2.29 and 1.20, respectively, to convert them to P_2_O_5_ and K_2_O. In the UK dataset, the application rates of some crops varied across seasons. In such cases, lacking intra-annual detail within our product, we used the season-averaged application rate to infer the annual average fertilizer application rate by crop. For the AU dataset and some countries in FUBC18-IDV and FUBC18-AGG with data spanning two calendar years, we allocated the reference year at the first calendar year. For datasets that provided the list of crops within a crop group, i.e., FUBC18-AGG and UK, the group application rate was assigned to all component crops in that group as per aggregations in Supplementary Table S3.

Other georeferenced and tabular corollary and benchmarking datasets (Table 2) were also harmonized to the same data format and administrative unit levels as the input datasets.

### Endogenous data quality indicators (Step 2)

We designed a multi-criteria ranking scheme to determine the best-fit value to represent fertilizer application rates for specific crops in subnational units where multiple sources were available across the eight selected input datasets. The ranking was based on three endogenous data quality indicators: *Q*_*c*_, crop specification; *Q*_*r*_, data resolution; and *Q*_*y*_, synchrony. Each indicator was assigned values between zero (lowest quality) and one (highest quality). For each dataset, the values of the indicators can vary across different crops and subnational units.

The *Q*_*c*_ indicator indicated whether the fertilizer data is specific to individual crops or crop aggregations as:

2 $$\begin{array}{ccc}{Q}_{c} & = & \left\{\begin{array}{cc}1 & \text{if crop} \mbox{-} \text{specific}\\ 0.5 & \text{if crop} \mbox{-} \text{aggregated}\end{array}\right.\end{array}$$

Dataset including both crop-specific and crop-aggregated data (e.g., UK) will have variable *Q*_*c*_ values across different crops, with a higher rank for individual crops.

The *Q*_*r*_ indicator ranked the administrative resolution of a dataset, with a higher rank given to datasets with a finer resolution as follows:

3 $${Q}_{r}=\left\{\begin{array}{c}\begin{array}{cc}1 & \text{if subnational}\\ 0.75 & \text{if national and subnational}\\ 0.5 & \text{if national}\end{array}\end{array}\right.$$

The *Q*_*y*_ indicator rated the synchrony level between the reference year *Y*_*r*_ of an input dataset and the reference year of NPKGRIDS, which was set to the period 2015–2020, henceforth referred to as ‘circa 2020’, and was defined as

4 $$\begin{array}{ccc}{Q}_{y} & = & \left\{\begin{array}{cc}\frac{{Y}_{r}-2000}{2015-2000} & \text{if}\,Y < 2015\\ 1 & \text{if}\,2015\le Y\le 2020\end{array}\right.\end{array}$$

*Q*_*y*_ increases as ${Y}_{r}$ approaches the 2015–2020 period and could vary in response to a wide range of reference years ${Y}_{r}$ within the same dataset e.g., HFUBC and US.

The endogenous data quality indicators defined above are summarized in Table 3 for all datasets used to compile NPKGRIDS. Operationally, we associated an average endogenous quality to each dataset *k*, crop *i* and subnational unit *r* as

5 $${Q}_{k,i,r}=\frac{{\left({Q}_{c}+{Q}_{r}+{Q}_{y}\right)}_{k,i,r}}{3}$$

**Table 3:** Value ranges of endogenous data quality indicators of all input datasets.

Dataset	Crop specification *Q*_*c*_	Data resolution *Q*_*r*_	Synchrony *Q*_*y*_
MFM	1	0.75	0
FUBC18-IDV	1	0.5	1
FUBC18-AGG	0.5	0.5	1
HFUCB	1	0.5	0.47–1
US	1	1	0.47–1
BY	1	1	1
UK	0.5–1	1	1
AU	1	1	1

### Global spatialization of fertilizer application rates (Step 3)

**Fig. 2:**
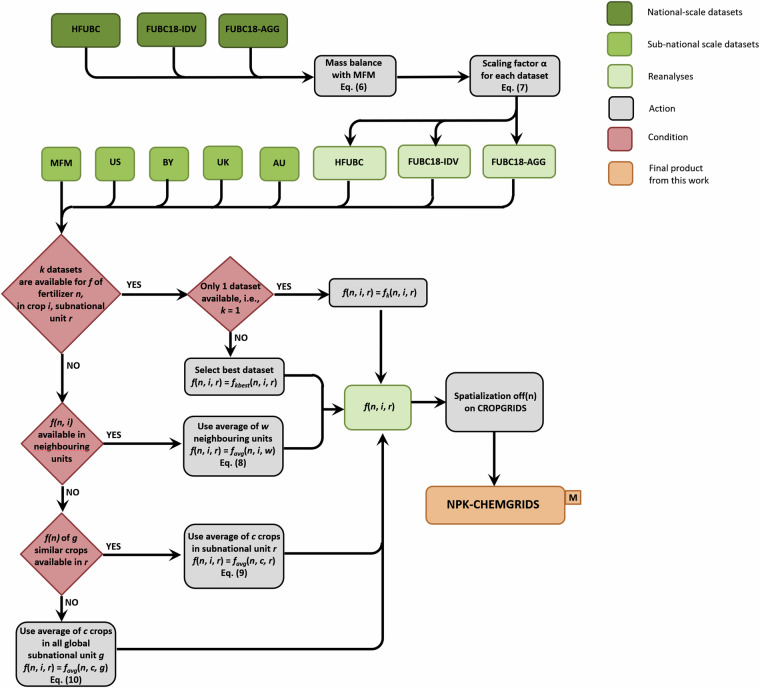
Algorithm for the assemblage of global maps of crop-specific fertilizer application rates. Refer to Tables 1, 2 for the names of the datasets.

An assemblage of global georeferenced application rates of N, P_2_O_5_ and K_2_O for individual crops was conducted following the algorithm in Fig. 2. First, we disaggregated the crop-specific national application rates of the three fertilizers in HFUCB, FUBC18-IDV and FUBC18-AGG into subnational application rates using the proportional allocations from national to sub-national level calculated from MFM, where MFM data is available. This step leads to the calculation of crop-specific application rate *f*_*k*_(*n*,*i*,*j*,*r*) for a dataset *k* of fertilizer *n* on crop *i* in subnational unit *r* of country *j* as

6 $${f}_{k}(n,i,j,r)=\alpha (n,i,j)\cdot {f}_{\text{MFM}}\left(n,i,j,r\right)$$

where *f*_MFM_ is the application rate of the corresponding crop and subnational unit in MFM and *α* is a scaling factor defined as

7 $$a(n,i,j)=\frac{{F}_{k}(n,i,j)\cdot {\sum }_{r|(i,j)}{A}_{{\rm{CR}}}(i,j,r)}{{\sum }_{r|(n,i,j)}[\,{f}_{{\rm{MFM}}}(n,i,j,r)\cdot {A}_{{\rm{CR}}}(i,j,r)\,]}$$

where *F*_*k*_(*n,i,j*) is the national-level application rate of fertilizer *n* in dataset *k* for crop *i* and country *j*, and *A*_CR_ is the corresponding crop harvested area in CROPGRIDS. In Eq. (7), we assumed that these proportions α were unchanged between 2000 and 2020. This is true only if such geographical differences were assumed to depend largely on agri-meteorological differences rather than management practices, or alternatively that geographical differences in the latter had remained similar over the two time periods.

For each fertilizer *n* applied to crop *i* in subnational unit *r*, we tested if the application rate was available from multiple datasets. If only one dataset *k* was available, the chosen application rate is *f*(*n,i,r*)  =  *f*_*k*_(*n,i,r*) (Fig. 2). If multiple datasets were available, we selected the best-fit dataset *k*_*best*_, which has the highest endogenous quality Q_*k,i,r*_ defined in Eq. (5), and thus, *f*(*n,i,r*)  =  ${f}_{{k}_{{best}}}$(*n,i,r*). If two datasets have equal Q_*k,i,r*_, the dataset with the most recent reference year was chosen as *k*_*best*_. Alternatively, if these datasets have the same reference year, ${f}_{{k}_{{best}}}\left(n,i,r\right)$ was calculated as the average value of all datasets with equal Q_*k,i,r*_ and reference year. If no datasets were available, we performed gap filling by first checking if data were available in the neighbouring subnational units. Specifically, if the application rates of fertilizer *n* on crop *i* were available in *w* bordering subnational units, the area-weighted average fertilizer application rate *f*_*avg*_(*n,i,w*) was computed over the *w* bordering subnational units as

8 $$f(n,i,r)={f}_{avg}(n,i,w)=\frac{{\sum }_{w|(n,i)}\,f(n,i,w)\cdot {n}_{w}}{\sum {n}_{w}}$$

with *n*_*w*_ being the number of shared-bordering grid cells. If there is no application rate for fertilizer *n* on crop *i* in neighbouring subnational units, we estimated *f*(*n,i,r*) from application rates on similar crops based on three criteria defined by FAO^39^: (a) classification (i.e., cereals, pulses, nuts, fruits and berries, spices, permanent oil-bearing crops, temporary oil-bearing crops, fodder crops, fibre crops, vegetables, and other permanent crops); (b) lifespan (i.e., temporary or permanent); and (c) stem type (i.e., herbaceous, shrubs or tree; see Supplementary Table 4). A crop *i* is considered similar to crops *c* if they share at least two of the three abovementioned criteria. If the application rate of fertilizer *n* on similar crops *c* were available within the subnational unit *r* (Fig. 2), we calculated *f*(*n,i,r*)  =  *f*_*avg*_(*n,c,r*), where *f*_*avg*_(*n,c,r*) is the area-weighted average fertilizer application rate across *c* similar crops in subnational unit *r*, such that

9 $$f(n,i,r)={f}_{avg}(n,c,r)=\frac{{\sum }_{c|(n,r)}\,f(n,c,r)\cdot {A}_{CR}(c,r)}{{\sum }_{c|(n,r)}{A}_{CR}(c,r)}$$

If there is no similar crop within the subnational unit *r* (Fig. 2), we computed *f*(*n,i,r*)  =  *f*_*avg*_(*n,c,g*), where *f*_*avg*_(*n,c,g*) is the area-weighted average application rate of *c* similar crops globally across all subnational units *g*, such that

10 $$f(n,i,r)={f}_{avg}(n,c,g)=\frac{{\sum }_{c|(n,g)}\,f(n,c,g)\cdot {A}_{CR}(c,g)}{{\sum }_{c|(n,g)}{A}_{CR}(c,g)}$$

**Fig. 3:**
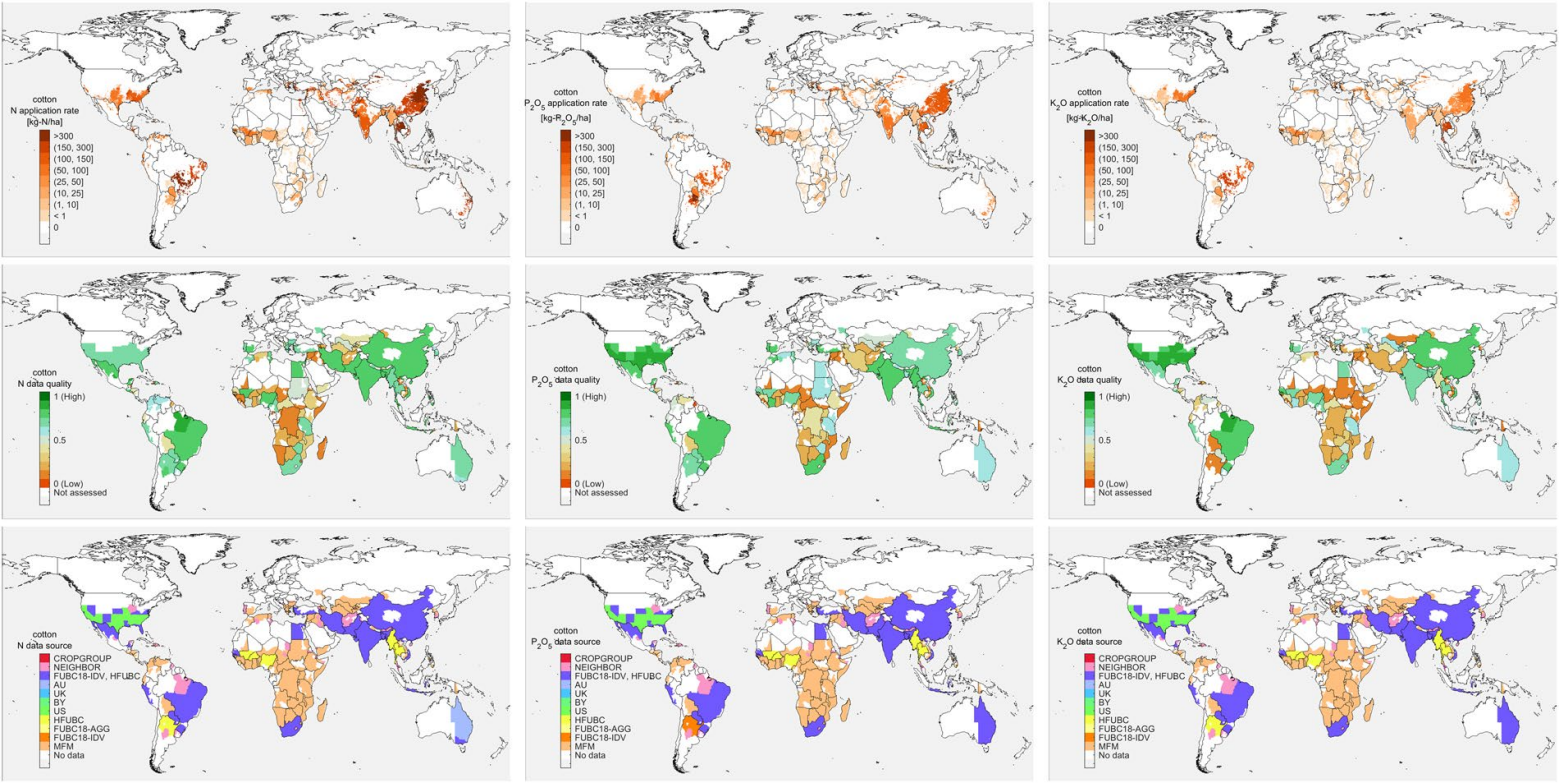
Example maps distributed in NPKGRIDS data for cotton. From left to right columns: N, P_2_O_5_, and K_2_O; from top to bottom rows: fertilizer application rate, data quality, and data source.

Finally, to construct the global georeferenced maps of fertilizer application rates by nutrients and crops, the subnational crop-specific fertilizer application rates were uniformly spatialized over the grid cells hosting crop *i* within that subnational unit using crop masks from CROPGRIDS. Example maps of N, P_2_O_5_ and K_2_O application rates for cotton, and the corresponding overall data quality and data sources used to construct the maps are shown in Fig. 3.

## Data Records

NPKGRIDS dataset distributes global georeferenced maps of N, P_2_O_5_, and K_2_O fertilizer application rates for 173 crops (refer to Supplementary Table 4 for the list of crops) for the year circa 2020 at a resolution of 0.05° (about 5.6 km at the equator) with a bounding box of −180° to 180° longitude and −90° and 90° latitude using the WGS-84 coordinate system. The georeferenced maps are distributed as NetCDF files, where grid cells containing ocean/water are marked as “-1”. Files included in the dataset are described in Table 4. NPKGRIDS dataset is available for public download from the *figshare* repository^40^ at 10.6084/m9.figshare.24616050. The data for P and K fertilizers are distributed in terms of oxide-based application rates. These can be converted to elemental-based application rates using the following conversions: 1 kg of P_2_O_5_ is equivalent to 0.436 kg of P, and 1 kg of K_2_O is equivalent to 0.83 kg of K.

**Table 4:** NPKGRIDS data distribution files and variables.

Folder Name	File Name	Description	Variable		
			Name	Description	Unit
NPKGRIDSv1.08_NC.zip	NPKGRIDSv1.08_YYYY.nc	Contains globally gridded data for crop YYYY.	Nrate	N application rate	kg-N ha^−1^
			Nqual	N data quality	—
			Nset	Best-fit dataset used for N	—
			P2O5rate	P_2_O_5_ application rate	kg-P_2_O_5_ ha^−1^
			P2O5qual	P_2_O_5_ data quality	—
			P2O5set	Best-fit dataset used for P_2_O_5_	—
			K2Orate	K_2_O application rate	kg-K_2_O ha^−1^
			K2Oqual	K_2_O data quality	—
			K2Oset	Best-fit dataset used for K_2_O	—
NPKGRIDSv1.08_PNG.zip	NPKGRIDSv1.08_YYYY.png	Images of N, P_2_O_5_, and K_2_O application rates of crop YYYY, and corresponding data quality and best-fit datasets.			
CODES.zip	MAIN.m, TABULATE_2_Patching.m, TABULATE_3_NEIGHBOR.m, TABULATE_4_CROPGROUP.m, TABULATE_5_FinalDataQuality.m	MATLAB scripts used to construct NPKGRIDS.			

All files are publicly available from *figshare* repository^40^.

## Technical Validation

### Validation of NPKGRIDS with national-level data from FAOSTAT and IFASTAT

Lacking additional datasets with fertilizers by crop data beyond those already used herein, we evaluated NPKGRIDS data using national-level total applications of N, P_2_O_5_, and K_2_O fertilizers provided by FAOSTAT^41^ (160 countries) and IFA^11^ (110 countries) (Table 1). To this end, we first calculated the total national-level applied mass *M(n,j)* of fertilizer *n* in country *j* estimated by NPKGRIDS as

11 $$M(n,j)={\sum }_{({p}{,}{i})|(n,{j})}{{A}}_{{\rm{CR}}}(p,{i}{,}{j})\cdot {f}(n,p,{i}{,}{j})$$

where *A*_CR_(*p*,*i*,*j*) is the harvested area of crop *i* in grid cell *p* of country *j* in CROPGRIDS^26^ and *f* is the corresponding application rate of fertilizer *n* in NPKGRIDS. The country boundaries were determined based on the GAUL^38^ dataset (level 0). We then compared *M(n,j)* against the corresponding fertilizer use reported in FAOSTAT and IFASTAT, *M*_FAO_ and *M*_IFA_, respectively, averaged over the 2015–2020 period. These comparisons were characterized using the coefficient of determination R^2^ (analogous to Nash-Sutcliffe efficiency coefficient), the concordance correlation coefficient (CCC), and the normalized root mean squared errors (NRMSE), expressed as

12 $${\text{R}}_{x}^{2}(n)=1-\frac{\sum _{j}{\left({O}_{x}(n,j)-E(n,j)\right)}^{2}}{\sum _{j}{\left({O}_{x}(n,j)-\bar{{O}_{x}}(n)\right)}^{2}}$$

13 $${{\rm{CCC}}}_{x}(n)=\frac{2\rho \left(n\right){\sigma }_{{O}_{x}}\left(n\right){\sigma }_{E}(n)}{{{\sigma }_{{O}_{x}}\left(n\right)}^{2}+{{\sigma }_{E}(n)}^{2}+{[\bar{{O}_{x}}(n)-\bar{E}(n)]}^{2}}$$

14 $${{\rm{NRMSE}}}_{x}(n)=\frac{\sqrt{\frac{\sum _{j}{\left[{M}_{x}(n,j)-M(n,j)\right]}^{2}}{{n}_{p}}}}{[{M}_{{\rm{x}},\max }\left(n\right)-{M}_{x,\min }\left(n\right)]}$$

where ${O}_{x}$ represents the logarithmic of either *M*_FAO_ or *M*_IFA_ and $E$ represents the logarithmic of national-level applied mass (*M*) calculated from NPKGRIDS. $\bar{{O}_{x}}$ and $\bar{E}$ are the corresponding means across all countries, ${{\sigma }_{{O}_{x}}}^{2}$ and ${{\sigma }_{E}}^{2}$ are the corresponding variances, and $\rho$ is the Pearson correlation coefficient between *O*_*x*_ and $E$. *M*_*x*_ represents either *M*_FAO_ or *M*_IFA_, ${M}_{x,\max }$ and ${M}_{x,\min }$ are the corresponding maximum and minimum fertilizer masses across all countries, and ${n}_{p}$ is the number of data points.

**Fig. 4:**
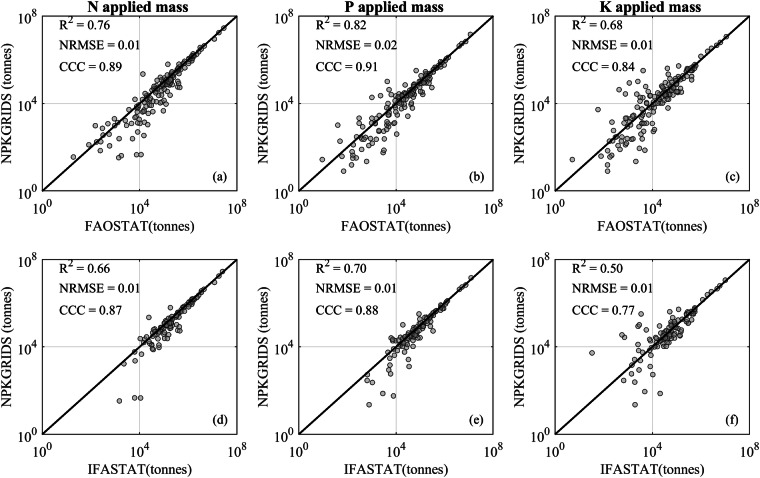
Comparison of fertilizer applied mass between NPKGRIDS and FAOSTAT (top row) and IFASTAT (bottom row) for N (left column), P_2_O_5_ (middle column), and K_2_O (right column). Each marker in the scatter plots represents a country and the black lines show the 1:1 ratio.

In NPKGRIDS, the global total N applied was 100 million tonnes, approximately 10% lower than the world estimates reported by FAOSTAT and IFASTAT for the year 2020, which stood at 110 and 112 million tonnes, respectively. At national level (Fig. 4, left column), the N applied mass calculated using NPKGRIDS matched relatively well with FAOSTAT (R^2^  =  0.76, CCC  =  0.89 and NRMSE = 0.01) and reasonably well with IFASTAT (R^2^ = 0.66, CCC = 0.87 and NRMSE = 0.01). In the comparison against FAOSTAT data, underestimation of N application was mostly identified in Africa, such as the Democratic Republic of Congo, Namibia, and Madagascar. NPKGRIDS consistently overestimated the N application in Iraq, Syria, and Jordan when comparing against FAOSTAT and IFASTAT data.

For phosphorus, NPKGRIDS estimated a global applied mass of 46 million tonnes, closely aligning with FAOSTAT’s and IFASTAT’s estimates for 2020, which were 48 and 49 million tonnes, respectively. The national-level comparisons for the total use of P_2_O_5_ had the strongest correlations with FAOSTAT (R^2^ = 0.82, CCC = 0.91 and NRMSE = 0.02) and IFASTAT (R^2^ = 0.70, CCC = 0.88 and NRMSE = 0.01, Fig. 4, middle column). Overall, and similarly to N data, discrepancies of P application data between NPKGRIDS and data from FAOSTAT and IFASTAT were more pronounced in Africa and in Middle Eastern countries.

For potassium, NPKGRIDS reported a global application of 40 million tonnes, matching well the global estimates by FAOSTAT and IFASTAT, which were 39 and 41 million tonnes, respectively. At the same time, the national-level comparisons of total K_2_O application showed less alignment with the FAO/IFA estimates (Fig. 4, right column), with lower correlations for both FAOSTAT (R^2^ = 0.68, CCC = 0.84 and NRMSE = 0.01) and IFASTAT (R^2^ = 0.50, CCC = 0.77 and NRMSE = 0.01). NPKGRIDS tended to overestimate K applied mass in North Africa and West Asia.

### Validation of NPKGRIDS with national and subnational data from NSOs

We obtained non-crop-specific total applied quantities of N, P_2_O_5_, and K_2_O at national and subnational levels for 37 countries and 166 subnational units from 2006 to 2020, including 32 countries in Europe^42^, India^43^, Pakistan^44^, China^45^, Iran^46^, and Sri Lanka^47^ (Table 2, Supplementary Table 2). Of the 37 countries, 11 countries provided subnational data while 26 countries provided only national-level data. Only 5 countries, with a total of 99 subnational units, provided K_2_O data. We calculated the 2015–2020 averages for all NSOs data, with the exception of Iran, for which the latest available data is from 2006. We aggregated pixel-level data in NPKGRIDS to national and subnational total applied masses of N, P_2_O_5_ and K_2_O following Eq. 11, with *j* being a country (level 0) or subnational (level 1) unit defined in the GAUL^38^ administrative unit boundaries. The quality of comparisons between NPKGRIDS and NSOs data was quantified using R^2^ (Eq. 12), CCC (Eq. 13) and NRMSE (Eq. 14).

**Fig. 5:**
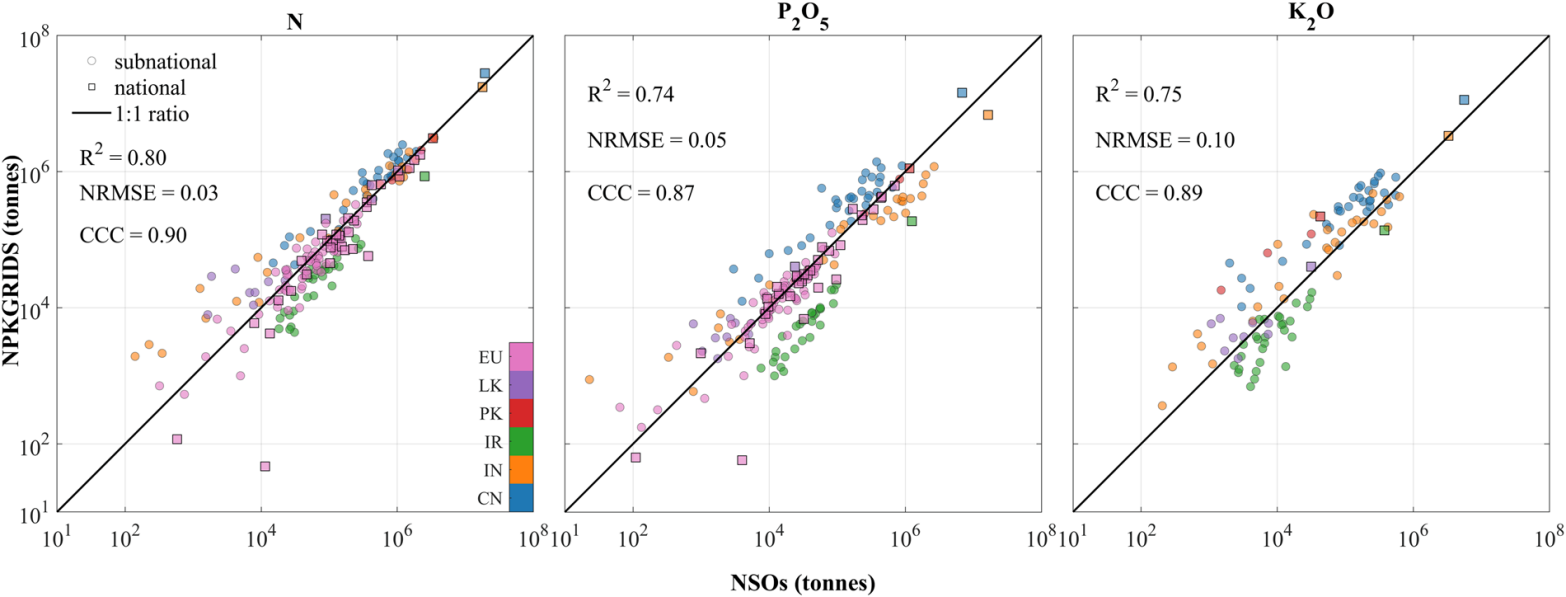
Comparison of national and subnational-level fertilizer masses used on all crops between NPKGRIDS and National Statistical Offices (NSOs). Total applied masses of (**a**) N, (**b**) P_2_O_5_, and (**c**) K_2_O. Coloured markers refer to various NSOs: EU (European Unions), LK (Sri Lanka), PK (Pakistan), IR (Iran), IN (India), and CN (China). Black lines show the 1:1 ratio.

The comparison of national and subnational levels N applied mass between NPKGRIDS and NSOs showed a relatively good agreement with R^2^ = 0.80, CCC = 0.90 and NRMSE = 0.03 (Fig. 5), while estimates for P_2_O_5_ and K_2_O were weaker, with R^2^ values of 0.74 and 0.75 against NSOs data, respectively.

The comparison of 32 countries against the statistical office of the European Union (EUROSTAT)^42^ data showed a good alignment at both national and subnational level, with a few exceptions. Specifically, total use of N and P_2_O_5_ fertilizers were significantly underestimated in Iceland and Ireland (N and P_2_O_5_) and in Malta (N). This underestimation was likely due to the high uncertainty in crop harvested area reported in CROPGRIDS for these countries, and for Ireland specifically, due to uncertainty fertilizer use on meadows and pastures. In Iceland, only potatoes were mapped in NPKGRIDS. In contrast, nutrient application in China was slightly overestimated.

### Limitations and uncertainty

NPKGRIDS incorporates uncertainties and errors of its input datasets, such as the source fertilizer datasets and the CROPGRIDS dataset used to spatially allocate the tabulated fertilizer application rates. Uncertainties can arise from errors in, or missing reporting of, fertilization amounts and crop area data submitted for national and international reporting. For example, MFM encountered data limitations in many lower- and middle-income countries and observed more anomalies in P and K fertilization data as compared to N. On the other hand, CROPGRIDS was constructed by reconciling multiple data sources, including surveys, remote sensing, and models, where each of these sources have uncertainties that will propagate into the construction of NPKGRIDS.

The spatialization of national and subnational-level data to grid cells implemented in NPKGRIDS introduces further uncertainty. For instance, the spatialization of national data (e.g., HFUBC, FUBC18-IDV, FUBC18-AGG) assumes that the relative ratio of fertilizer usage within a country follows the same patterns observed in MFM (Eq. 7), ignoring potential relative changes in cropping practices that may have occurred across different subnational units within a country. Additionally, for information taken directly from MFM, changes in fertilizer usage that may have occurred in those regions over the past 20 years are not considered.

Finally, NPKGRIDS excluded some small countries and territories due to constraint in spatial resolution, including Falkland, Faroe Islands, French Southern and Antarctic Territories (SAT), Heart Island, Isle of Man, Kingman Reef, Kiribati, Ma’tan al-Sarra, Mayotte, Netherland Antilles, Palau, Réunion, Saint Pierre, South Georgia, Svalbard, and Virgin Islands.

### Data quality of NPKGRIDS

To quantify the underlying uncertainty, we computed a data quality indicator at subnational unit level based on endogenous quality indicators and comparisons against FAOSTAT and IFASTAT data. The overall data quality $Q(n,i,j,r)$ of NPKGRIDS for nutrient *n* (i.e., N, P_2_O_5_, and K_2_O), crop *i* in subnational unit *r* in country *j* is computed as

15 $$Q(n,i,j,r)=\frac{{Q}_{\text{k}}(n,i,j,r)+{Q}_{\text{FAO}}(n,j)+{Q}_{\text{IFA}}(n,j)}{3}$$

where *Q*_*k*_ is the endogenous quality of the chosen dataset calculated using Eq. (5), and the qualities of benchmarking *Q*_*FAO*_ and *Q*_*IFA*_ against FAOSTAT^41^ and IFASTAT^11^ datasets are defined as

16 $${Q}_{\text{x}}(n,j)=1-\min \left\{1,\frac{\left|M(n,j)-{\text{M}}_{x}(n,j)\right|}{\,{\text{M}}_{\text{x}}(n,j)}\right\}$$

with *x* being either FAO or IFA and *Q*_*x*_ having values between 0 (low quality) and 1 (high quality). For those subnational units where the application rates were gap-filled, we assigned zero to the corresponding *Q*_*k*_. Maps of data quality are distributed along with NPKGRIDS dataset. Examples of data quality maps for cotton are shown in Fig. 3 (second row).

## Usage Notes

All georeferenced maps distributed in NPKGRIDS dataset^40^ are formatted as standard NetCDF4 files. Various coding languages (e.g., MATLAB, Python, Julia, R) and software (ArcGIS, QGIS, Panoply) can be used to read and analysis these files. NPKGRIDS dataset includes the same crops as CROPGRIDS^26^ dataset, which follow the naming system used by FAO^28^.

## Supplementary information

Supplementary Information

## Data Availability

All computational work in this study, including data processing and validation, were conducted using MATLAB version R2022b. Main codes used to construct NPKGRIDS are distributed in the “CODES.zip” folder (Table 4) along with NPKGRIDS dataset available for public download from the figshare repository^40^ at 10.6084/m9.figshare.24616050.
