# Supplementary material for: NPKGRIDS: a global georeferenced dataset of N, P2O5, and K2O fertilizer application rates for 173 crops
Source: Sci Data. 2024 Oct 30;11:1179. doi: 10.1038/s41597-024-04030-4 (PMC11526156; doi:10.1038/s41597-024-04030-4)
Supplement: Supplementary file 1 — Supplementary Information [file 41597_2024_4030_MOESM1_ESM.pdf]

Supplementary Information

NPKGRIDS: a global georeferenced dataset of N, P_2O_5, and K_2O fertilizer application rates for 173 crops

Thu Ha Nguyen[1,2], Fiona H. M. Tang[3,4], Giulia Conchedda[5], Leon Casse[1,6], Griffiths Obli-Laryea[6], Francesco N. Tubiello[6], Federico Maggi[1,7]

[1]Environmental Engineering, School of Civil Engineering, The University of Sydney, Sydney, New South Wales, Australia.
[2]Alluvium Consulting, Sydney, New South Wales, Australia.
[3]School of Environmental and Rural Science, University of New England, Armidale, New South Wales 2351, Australia.
[4]Department of Civil Engineering, Monash University, Clayton, Victoria, Australia.
[5]Land and Water Division, Food and Agriculture Organization of the United Nations, Viale delle Terme di Caracalla, Rome 00153, Italy.
[6]Statistics Division, Food and Agriculture Organization of the United Nations, Viale delle Terme di Caracalla, Rome 00153, Italy.
[7]Sydney Institute of Agriculture, The University of Sydney, Sydney, NSW, 2006, Australia

Correspondence to: Federico Maggi (federico.maggi@sydney.edu.au)

Table of Contents:

**Supplementary Tables**		
Table 1	Matching and aggregation of crop names for NPKGRIDS and the selected crop-specific fertilization input datasets	2
Table 2	Descriptions of fertilization data obtained from various national statistical bureaus	3
Table 3	Details of crop aggregations applied to individual crops	4
Table 4	Details of crop type classifications	5-7
Table 5	The selected best-fit datasets used to construct the global maps of N application rates of each individual crop	8-11
Table 6	The selected best-fit datasets used to construct the global maps of P_2O_5 application rates of each individual crop	12-15
Table 7	The selected best-fit datasets used to construct the global maps of K_2O application rates of each individual crop	16-19

Supplementary Tables

Supplementary Table 1 Matching and aggregation of crop names for NPKGRIDS and the selected crop-specific fertilization datasets. Names in brackets refer to the names used in the original dataset and corresponding aggregation.

Datasets	Crop names in MRF dataset
NPKGRIDS	abaca, agave, alfalfa, almond, aniseetc, apple, apricot, areca, artichoke, asparagus, avocado, bambara, banana, barley, bean, beetfor, berrynes, blueberry, brazil, broadbean, buckwheat, cabbage, cabbagefor, canaryseed, carob, carrot, carrotfor, cashew, cashewapple, cassava, castor, cauliflower, cerealnes, cherry, chestnut, chickpea, chicory, chilleetc, cinnamon, citrusnes, clove, clover, cocoa, coconut, coffee, cotton, cowpea, cranberry, cucumberetc, currant, date, eggplant, fibrenes, fig, flax, fonio, fornes, fruitnes, garlic, ginger, gooseberry, grape, grapefruitetc, grassnes, greenbean, greenbroadbean, greencorn, greenonion, greenpea, groundnut, hazelnut, hemp, hempseed, hop, jute, jutelikefiber, kapokfiber, kapokseed, karite, kiwi, kolanut, legumenes, lemonlime, lentil, lettuce, linseed, lupin, maize, maizefor, mango, mate, melonetc, melonseed, millet, mixedgrain, mixedgrass, mushroom, mustard, nutmeg, nutnes, oats, oilpalm, oilseedfor, oilseednes, okra, olive, onion, orange, papaya, pea, peachetc, pear, pepper, peppermint, persimmon, pigeonpea, pimento, pineapple, pistachio, plantain, plum, popcorn, poppy, potato, pulsenes, pumpkinetc, pyrethrum, quince, quinoa, ramie, rapeseed, rasberry, rice, rootnes, rubber, rye, ryefor, safflower, sesame, sisal, sorghum, sorghumfor, sourcherry, soybean, spicenes, spinach, stonefruitnes, strawberry, stringbean, sugarbeet, sugarcane, sugarnes, sunflower, swedefor, sweetpotato, tangetc, taro, tea, tobacco, tomato, triticale, tropicalnes, tung, turnipfor, vanilla, vegetablenes, vegfor, vetch, walnut, watermelon, wheat, yam, yautia
MFM	alfalfa, almond, apple, apricot, artichoke, asparagus, avocado, banana, barley, bean, beetfor, berrynes, blueberry, broadbean, buckwheat, cabbage, cabbagefor, canaryseed, carob, carrot, cashewapple, cassava, castor, cauliflower, cerealnes, cherry, chestnut, chickpea, chilleetc (chilietc), citrusnes, clover, cocoa, coconut, coffee, cotton, cowpea, cranberry, cucumberetc, currant, date, eggplant, fig, flax, fornes, fruitnes, garlic, gooseberry, grape, grapefruitetc, grassnes, greenbean, greenbroadbean, greencorn, greenonion, greenpea, groundnut, hazelnut, hemp, hempseed, hop, jute, jutelikefiber, kiwi, legumenes, lemonlime, lentil, lettuce, linseed, lupin, maize, maizefor, mango, melonetc, millet, mixedgrain, mixedgrass, mushroom, mustard, nutnes, oats, oilpalm, oilseedfor, oilseednes, okra, olive, onion, orange, papaya, pea, peachetc, pear, pepper, persimmon, pigeonpea, pineapple, pistachio, plantain, plum, poppy, potato, pulsenes, pumpkinetc, quince, rapeseed, rasberry, rice, rootnes, rubber, rye, ryefor, safflower, sesame, sisal, sorghum, sorghumfor, sourcherry, soybean, spinach, stonefruitnes, strawberry, stringbean, sugarbeet, sugarcane, sunflower, sweetpotato, tangetc, taro, tea, tobacco, tomato, triticale, tropicalnes, turnipfor, vegetablenes, vegfor, walnut, watermelon, wheat, yam
HFUBC	coffee, cotton, maize (grain maize), maizefor (maize green), oilpalm, rapeseed, rice, soybean, surgarbeet, sugarcane, tea, wheat
FUBC18-IDV	alfalfa (lucerne), banana, barley, bean, cassava, cocoa, coconut, coffee, cotton, cowpea, flax (flax fiber), fonio, garlic, ginger, grape (vineyards), greencorn (sweet corn), groundnut (peanut), jute, maize (grain maize), maizefor (green maize, maize for biogas, silage maize), melonetc (food melons, including for seed), millet, oilpalm, olive, onion, orange, pineapple, potato, rapeseed (rapeseed/canola, oilseed rape), rice, rubber, sesame, sorghum, soybean (soya beans), sugarbeet, sugarcane, sunflower (sunflower seeds), taro (cocoyam/taro), tea, tobacco, tomato (tomato – industrial), wheat, yam
US	cotton, maize (corn), soybean (soybeans), wheat
BY	flax, potato (potatoes), surgarbeet (sugar beets)
UK	barley (spring barley + winter barley), bean (field beans (harvested dry)), greenpea (vining peas (for human consumption)), linseed, maizefor (forage maize), oats, pea (field peas (harvested dry)), potato (potatoes (maincrop)), rapeseed (winter oilseed rape + spring oilseed rape), sugarbeet (sugar beet), wheat (winter wheat + spring wheat)
AU	cotton, rice, sugarcane

Supplementary Table 2 Descriptions of fertilization data obtained from various national statistical bureaus.

Country name	Number of subnational units	Fertilizer	Temporal coverage	Data link
Albania	0	N, P	2015 - 2020	https://ec.europa.eu/eurostat/databrowser/view/AEI_FM_USEFERT__custom_3050970/
Austria	0	N, P	2015 - 2020	as above
Belgium	0	N, P	2015 - 2020	as above
Bulgaria	0	N, P	2015 - 2020	as above
China	31	N, P_2O_5, K_2O	2019	https://data.stats.gov.cn/english/easyquery.htm?cn=C01
Croatia	0	N, P	2015 - 2020	https://ec.europa.eu/eurostat/databrowser/view/AEI_FM_USEFERT__custom_3050970/
Cyprus	0	N, P	2015 - 2020	as above
Czech Republic	5	N, P	2015 - 2020	as above
Denmark	0	N, P	2015 - 2020	as above
Estonia	0	N, P	2015 - 2020	as above
Finland	4	N, P	2015 - 2020	as above
France	22	N, P	2015 - 2020	as above
Germany	0	N, P	2015 - 2020	as above
Greece	0	N, P	2015 - 2020	as above
Hungary	0	N, P	2015 - 2020	as above
Iceland	0	N, P	2015 - 2020	as above
India	28	N, P, K	2017 - 2019	https://foodprocessingindia.gov.in/publication
Iran	28	N, P_2O_5, K_2O	2006	https://irandataportal.syr.edu/agriculture-rural-development
Ireland	0	N, P	2015 - 2020	https://ec.europa.eu/eurostat/databrowser/view/AEI_FM_USEFERT__custom_3050970/
Italy	0	N, P	2015 - 2020	as above
Latvia	0	N, P	2015 - 2020	as above
Lithuania	0	N, P	2015 - 2020	as above
Luxembourg	0	N, P	2015 - 2020	as above
Malta	0	N, P	2015 - 2020	as above
Netherlands	0	N, P	2015 - 2020	as above
Norway	0	N, P	2015 - 2020	as above
Pakistan	3	N, P_2O_5, K_2O	2015 - 2019	https://www.pbs.gov.pk/agriculture-statistics-tables
Poland	16	N, P	2015 - 2020	https://ec.europa.eu/eurostat/databrowser/view/AEI_FM_USEFERT__custom_3050970/
Portugal	0	N, P	2015 - 2020	as above
Romania	0	N, P	2015 - 2020	as above
Slovakia	0	N, P	2015 - 2020	as above
Slovenia	0	N, P	2015 - 2020	as above
Spain	17	N, P	2015 - 2020	as above
Sri Lanka	9	N, P_2O_5, K_2O	2018	https://doa.gov.lk/naicc-books/#1628491951058-49ca7ba3-ac39
Sweden	0	N, P	2015 - 2020	https://ec.europa.eu/eurostat/databrowser/view/AEI_FM_USEFERT__custom_3050970/
Switzerland	0	N, P	2015 - 2020	as above
Turkey	3	N, P	2015 - 2020	as above

Supplementary Table 3 Details of crop aggregations applied to individual crops

Datasets	Crop aggregations	Applied individual crops	Applied countries
FUBC18-AGG	Fruits (Pineapple + Mangoe + Longan + Rambutan + Durian + Mangosteen + Lychee + Tangerine + Longkong + Lime) & Treenuts	lemonlime, mango, pineapple, tangetc	Thailand
	Horticulture (cabbages, spinach, carrots, tomatoes, melon, bulb onions and pineapple)	cabbage, carrot, greenonion, melonetc, pineapple, spinach, tomato	Tanzania
	Maize, Total	maize, maizefor	China, USA
	Mil - Sorghum	millet, sorghum	Mali
	Permanent crops (fruit, vineyard)	grape	Austria, Belgium, Bulgaria, Croatia, Cyprus, Czech Republic, Denmark, Estonia, Finland, France, Germany, Greece, Italy, Latvia, Lithuania, Netherlands, Poland, Portugal, Romania, Slovakia, Slovenia, Spain, Sweden, UK
	Rye, triticale, oats, rice	oats, rice, rye, triticale	Austria, Belgium, Bulgaria, Cyprus, Czech Republic, Denmark, Estonia, Finland, France, Germany, Greece, Hungary, Ireland, Italy, Latvia, Lithuania, Netherlands, Norway, Poland, Portugal, Romania, Slovakia, Slovenia, Spain, Sweden, UK
	Sugar beet and Sugar cane	sugarbeet, sugarcane	Egypt, Iran, Mexico, Morocco
	Sunflower, soya, linseed	linseed, soybean, sunflower	Austria, Belgium, Bulgaria, Croatia, Czech Republic, Finland, France, Germany, Greece, Hungary, Italy, Latvia, Lithuania, Netherlands, Poland, Portugal, Romania, Slovakia, Slovenia, Spain, Sweden, UK
	Roots & Tubers (Potato & Sweet potato)	potato, sweetpotato	China
	Fruits (apples, citrus, oranges)	apple, orange	China
	Vegetables (Garlic+Shallot+Onion+Potato+Tomato+ Baby corn+Pepper)	garlic, greenonion, onion, pepper, potato, tomato	Thailand
	Other Oil Crops (including rapeseed)	rapeseed	Australia, Iran, Mexico, Russia, Vietnam
UK	Rye/triticale/Durum wheat	rye, triticale	UK

Supplementary Table 4 Details of crop type classifications

Crop name	Crop Classification	Lifespan	Stem type
abaca	Other permanent crops	Permanent (P)	Shrubs
agave	Other permanent crops	P	Shrubs
alfalfa	Fodder crops (Temporary and permanent)	Temporary (T)	Herb
almond	Nuts	P	Trees
aniseetc	Spices, condiments and aromatic herbs	T	Herb
apple	Fruits and berries	P	Trees
apricot	Fruits and berries	P	Trees
areca	Oil-bearing crops (Permanent only)	P	Trees
artichoke	Vegetables	T	Herb
asparagus	Vegetables	T	Herb
avocado	Fruits and berries	P	Trees
bambara	Pulses	T	Herb
banana	Fruits and berries	P	Shrubs
barley	Cereals	T	Herb
bean	Pulses	T	Herb
beetfor	Fodder crops (Temporary and permanent)	T	Herb
berrynes	Fruits and berries	P	Shrubs
blueberry	Fruits and berries	P	Shrubs
brazil	Nuts	P	Trees
broadbean	Pulses	T	Herb
buckwheat	Cereals	T	Herb
cabbage	Vegetables	T	Herb
cabbagefor	Fodder crops (Temporary and permanent)	T	Herb
canaryseed	Cereals	T	Herb
carob	Fruits and berries	P	Trees
carrot	Vegetables	T	Herb
carrotfor	Fodder crops (Temporary and permanent)	T	Herb
cashew	Nuts	P	Trees
cashewapple	Fruits and berries	P	Trees
cassava	Roots and tubers	T	Herb
castor	Oil-bearing crops (Temporary only)	T	Herb
cauliflower	Vegetables	T	Herb
cerealnes	Cereals	T	Herb
cherry	Fruits and berries	P	Trees
chestnut	Nuts	P	Trees
chickpea	Pulses	T	Herb
chicory	Vegetables	T	Herb
chilleetc	Vegetables	T	Herb
cinnamon	Spices, condiments and aromatic herbs	P	Trees
citrusnes	Fruits and berries	P	Trees
clove	Spices, condiments and aromatic herbs	P	Trees
clover	Fodder crops (Temporary and permanent)	T	Herb
cocoa	Other permanent crops	P	Shrubs
coconut	Oil-bearing crops (Permanent only)	P	Trees
coffee	Other permanent crops	P	Shrubs
cotton	Fibre crops (Temporary only)	T	Herb
cowpea	Pulses	T	Herb
cranberry	Fruits and berries	P	Shrubs
cucumberetc	Vegetables	T	Herb
currant	Fruits and berries	P	Shrubs
date	Fruits and berries	P	Trees
eggplant	Vegetables	T	Herb
fibrenes	Other permanent crops	P	Shrubs
fig	Fruits and berries	P	Trees
flax	Fibre crops (Temporary only)	T	Herb
fonio	Cereals	T	Herb
fornes	Fodder crops (Temporary and permanent)	T	Herb
fruitnes	Fruits and berries	P	Trees
garlic	Vegetables	T	Herb
ginger	Spices, condiments and aromatic herbs	P	Herb

gooseberry	Fruits and berries	P	Shrubs
grape	Fruits and berries	P	Shrubs
grapefruitetc	Fruits and berries	P	Trees
grassnes	Fodder crops (Temporary and permanent)	T	Herb
greenbean	Vegetables	T	Herb
greenbroadbean	Vegetables	T	Herb
greencorn	Vegetables	T	Herb
greenonion	Vegetables	T	Herb
greenpea	Vegetables	T	Herb
groundnut	Oil-bearing crops (Temporary only)	T	Herb
hazelnut	Nuts	P	Trees
hemp	Fibre crops (Temporary only)	T	Herb
hempseed	Oil-bearing crops (Temporary only)	T	Herb
hop	Other permanent crops	P	Herb
jute	Fibre crops (Temporary only)	T	Herb
jutelikefiber	Fibre crops (Temporary only)	T	Herb
kapokfiber	Oil-bearing crops (Permanent only)	P	Trees
kapokseed	Oil-bearing crops (Permanent only)	P	Trees
karite	Oil-bearing crops (Permanent only)	P	Trees
kiwi	Fruits and berries	P	Shrubs
kolanut	Nuts	P	Trees
legumenes	Pulses	T	Herb
lemonlime	Fruits and berries	P	Trees
lentil	Pulses	T	Herb
lettuce	Vegetables	T	Herb
linseed	Oil-bearing crops (Temporary only)	T	Herb
lupin	Pulses	T	Herb
maize	Cereals	T	Herb
maizefor	Fodder crops (Temporary and permanent)	T	Herb
mango	Fruits and berries	P	Trees
mate	Other permanent crops	P	Shrubs
melonetc	Vegetables	T	Herb
melonseed	Oil-bearing crops (Temporary only)	T	Herb
millet	Cereals	T	Herb
mixedgrain	Cereals	T	Herb
mixedgrass	Fodder crops (Temporary and permanent)	T	Herb
mushroom	Vegetables	T	Herb
mustard	Oil-bearing crops (Temporary only)	T	Herb
nutmeg	Spices, condiments and aromatic herbs	P	Trees
nutnes	Nuts	P	Trees
oats	Cereals	T	Herb
oilpalm	Oil-bearing crops (Permanent only)	P	Trees
oilseedfor	Fodder crops (Temporary and permanent)	T	Herb
oilseednes	Oil-bearing crops (Temporary only)	T	Herb
okra	Vegetables	T	Herb
olive	Oil-bearing crops (Permanent only)	P	Trees
onion	Vegetables	T	Herb
orange	Fruits and berries	P	Trees
papaya	Fruits and berries	P	Trees
pea	Pulses	T	Herb
peachetc	Fruits and berries	P	Trees
pear	Fruits and berries	P	Trees
pepper	Spices, condiments and aromatic herbs	P	Shrubs
peppermint	Spices, condiments and aromatic herbs	T	Herb
persimmon	Fruits and berries	P	Trees
pigeonpea	Pulses	T	Herb
pimento	Spices, condiments and aromatic herbs	P	Trees
pineapple	Fruits and berries	P	Shrubs
pistachio	Nuts	P	Trees
plantain	Fruits and berries	P	Shrubs
plum	Fruits and berries	P	Trees
popcorn	Cereals	T	Herb
poppy	Oil-bearing crops (Temporary only)	T	Herb
potato	Roots and tubers	T	Herb

pulsenes	Pulses	T	Herb
pumpkinetc	Vegetables	T	Herb
pyrethrum	Other Crops	T	Shrubs
quince	Fruits and berries	P	Trees
quinoa	Cereals	T	Herb
ramie	Fibre crops (Temporary only)	P	Herb
rapeseed	Oil-bearing crops (Temporary only)	T	Herb
rasberry	Fruits and berries	P	Shrubs
rice	Cereals	T	Herb
rootnes	Roots and tubers	T	Herb
rubber	Other permanent crops	P	Trees
rye	Cereals	T	Herb
ryefor	Fodder crops (Temporary and permanent)	T	Herb
safflower	Oil-bearing crops (Temporary only)	T	Herb
sesame	Oil-bearing crops (Temporary only)	T	Herb
sisal	Other permanent crops	P	Herb
sorghum	Cereals	T	Herb
sorghumfor	Fodder crops (Temporary and permanent)	T	Herb
sourcherry	Fruits and berries	P	Trees
soybean	Oil-bearing crops (Temporary only)	T	Herb
spicenes	Spices, condiments and aromatic herbs	P	Trees
spinach	Vegetables	T	Herb
stonefruitnes	Fruits and berries	P	Trees
strawberry	Fruits and berries	P	Shrubs
stringbean	Vegetables	T	Herb
sugarbeet	Sugar crops	T	Herb
sugarcane	Sugar crops	T	Herb
sugarnes	Sugar crops	T	Herb
sunflower	Oil-bearing crops (Temporary only)	T	Herb
swedefor	Fodder crops (Temporary and permanent)	T	Herb
sweetpotato	Roots and tubers	T	Herb
tangetc	Fruits and berries	P	Trees
taro	Roots and tubers	T	Herb
tea	Other permanent crops	P	Shrubs
tobacco	Tobacco	T	Herb
tomato	Vegetables	T	Herb
triticale	Cereals	T	Herb
tropicalnes	Fruits and berries	P	Trees
tung	Oil-bearing crops (Permanent only)	P	Trees
turnipfor	Fodder crops (Temporary and permanent)	T	Herb
vanilla	Spices, condiments and aromatic herbs	P	Shrubs
vegetablenes	Fodder crops (Temporary and permanent)	T	Herb
vegfor	Vegetables	T	Herb
vetch	Pulses	T	Herb
walnut	Nuts	P	Trees
watermelon	Vegetables	T	Herb
wheat	Cereals	T	Herb
yam	Roots and tubers	T	Herb
yautia	Roots and tubers	T	Herb

Supplementary Table 5 The selected best-fit datasets used to construct the global maps of N application rates of each individual crop.

Crops	Applied Mass [tonnes]	Datasets selected [% contribution of the dataset to the global map]
abaca	2010	CROPGROUP [100%]
agave	3095	CROPGROUP [100%]
alfalfa	345295	MFM [96.274%], FUBC18-IDV [0.219%], NEIGHBOR [3.507%]
almond	84076	MFM [99.774%], NEIGHBOR [0.226%]
aniseetc	158934	CROPGROUP [100%]
apple	721575	MFM [26.401%], FUBC18-AGG [72.929%], NEIGHBOR [0.670%]
apricot	30130	MFM [99.518%], NEIGHBOR [0.482%]
areca	24783	CROPGROUP [100%]
artichoke	15045	MFM [98.919%], NEIGHBOR [1.081%]
asparagus	253169	MFM [99.946%], NEIGHBOR [0.054%]
avocado	67619	MFM [99.999%], NEIGHBOR [0.001%]
bambara	272	CROPGROUP [100%]
banana	561797	MFM [96.895%], FUBC18-IDV [1.263%], NEIGHBOR [1.842%]
barley	2507209	MFM [47.204%], FUBC18-IDV [45.948%], GB [6.428%], NEIGHBOR [0.420%]
bean	756647	MFM [99.323%], FUBC18-IDV [0.527%], NEIGHBOR [0.150%]
beetfor	8911	MFM [99.283%], NEIGHBOR [0.717%]
berrynes	6613	MFM [98.687%], NEIGHBOR [1.313%]
blueberry	4535	MFM [92.828%], NEIGHBOR [7.172%]
brazil	7	CROPGROUP [100%]
broadbean	86958	MFM [98.854%], NEIGHBOR [1.146%]
buckwheat	58749	MFM [99.981%], NEIGHBOR [0.019%]
cabbage	293805	MFM [99.907%], FUBC18-AGG [0.001%], NEIGHBOR [0.093%]
cabbagefor	5350	MFM [68.384%], NEIGHBOR [31.616%]
canaryseed	7964	MFM [99.845%], NEIGHBOR [0.155%]
carob	6839	MFM [99.979%], NEIGHBOR [0.021%]
carrot	127700	MFM [99.910%], FUBC18-AGG [0.000%], NEIGHBOR [0.090%]
carrotfor	24	CROPGROUP [100%]
cashew	32461	CROPGROUP [100%]
cashewapple	4849	MFM [99.981%], NEIGHBOR [0.019%]
cassava	246052	MFM [45.795%], FUBC18-IDV [52.837%], NEIGHBOR [1.368%]
castor	36094	MFM [99.955%], NEIGHBOR [0.045%]
cauliflower	182827	MFM [99.922%], NEIGHBOR [0.078%]
cerealnes	50215	MFM [97.120%], NEIGHBOR [2.880%]
cherry	19951	MFM [99.991%], NEIGHBOR [0.009%]
chestnut	29282	MFM [99.950%], NEIGHBOR [0.050%]
chickpea	101847	MFM [98.740%], NEIGHBOR [1.260%]
chicory	1418	CROPGROUP [100%]
chilleetc	185947	CROPGROUP [100%]
cinnamon	22310	CROPGROUP [100%]
citrusnes	69484	MFM [96.793%], NEIGHBOR [3.207%]
clove	41744	CROPGROUP [100%]
clover	26217	MFM [91.941%], NEIGHBOR [8.059%]
cocoa	175441	MFM [93.699%], FUBC18-IDV [6.119%], NEIGHBOR [0.182%]
coconut	69516	MFM [72.784%], FUBC18-IDV [23.269%], NEIGHBOR [3.947%]
coffee	867897	MFM [49.734%], FUBC18-IDV [0.000%], HFUBC [0.537%], FUBC18-IDV & HFUBC [49.651%], NEIGHBOR [0.078%]
cotton	4030991	MFM [5.126%], HFUBC [2.023%], US [6.983%], AU [0.580%], FUBC18-IDV & HFUBC [85.070%], NEIGHBOR [0.218%]
cowpea	12499	MFM [53.955%], FUBC18-IDV [31.900%], NEIGHBOR [14.145%]
cranberry	1575	MFM [99.883%], NEIGHBOR [0.117%]
cucumberetc	278441	MFM [99.921%], NEIGHBOR [0.079%]
currant	5767	MFM [99.992%], NEIGHBOR [0.008%]
date	167718	MFM [99.778%], NEIGHBOR [0.222%]
eggplant	249705	MFM [99.607%], NEIGHBOR [0.393%]
fibrenes	4058	CROPGROUP [100%]
fig	15754	MFM [98.689%], NEIGHBOR [1.311%]
flax	28838	MFM [92.752%], BY [0.698%], NEIGHBOR [6.550%]
fonio	12410	FUBC18-IDV [0.841%], NEIGHBOR [99.159%]

fornes	413658	MFM [99.092%], NEIGHBOR [0.908%]
fruitnes	184911	MFM [99.783%], NEIGHBOR [0.217%]
garlic	215217	MFM [98.558%], FUBC18-IDV [0.177%], FUBC18-AGG [1.066%], NEIGHBOR [0.199%]
ginger	9330	FUBC18-IDV [22.861%], NEIGHBOR [77.139%]
gooseberry	1226	MFM [99.976%], NEIGHBOR [0.024%]
grape	459349	MFM [67.105%], FUBC18-IDV [1.198%], FUBC18-AGG [31.371%], NEIGHBOR [0.327%]
grapefruitetc	48593	MFM [99.952%], NEIGHBOR [0.048%]
grassnes	46508	MFM [83.359%], NEIGHBOR [16.641%]
greenbean	194196	MFM [99.442%], NEIGHBOR [0.558%]
greenbroadbean	11404	MFM [99.991%], NEIGHBOR [0.009%]
greencorn	124323	MFM [67.258%], FUBC18-IDV [4.569%], HFUBC [8.329%], NEIGHBOR [19.844%]
greenonion	24935	MFM [92.659%], NEIGHBOR [7.341%]
greenpea	333674	MFM [99.743%], NEIGHBOR [0.257%]
groundnut	737487	MFM [94.044%], FUBC18-IDV [5.941%], NEIGHBOR [0.016%]
hazelnut	80865	MFM [99.854%], NEIGHBOR [0.146%]
hemp	4553	MFM [87.844%], NEIGHBOR [12.156%]
hempseed	19	MFM [99.669%], NEIGHBOR [0.331%]
hop	8036	MFM [99.245%], NEIGHBOR [0.755%]
jute	96763	MFM [55.279%], FUBC18-IDV [44.708%], NEIGHBOR [0.013%]
jutelikefiber	19671	MFM [99.885%], NEIGHBOR [0.115%]
kapokfiber	27781	CROPGROUP [100%]
kapokseed	15588	CROPGROUP [100%]
karite	2003	CROPGROUP [100%]
kiwi	6023	MFM [91.112%], NEIGHBOR [8.888%]
kolanut	539	CROPGROUP [100%]
legumenes	22146	MFM [97.728%], NEIGHBOR [2.272%]
lemonlime	118405	MFM [99.263%], FUBC18-AGG [0.440%], NEIGHBOR [0.297%]
lentil	128456	MFM [99.768%], NEIGHBOR [0.232%]
lettuce	185643	MFM [99.754%], NEIGHBOR [0.246%]
linseed	40266	MFM [85.260%], FUBC18-AGG [7.091%], GB [5.217%], NEIGHBOR [2.433%]
lupin	14773	MFM [98.705%], NEIGHBOR [1.295%]
maize	20786917	MFM [2.507%], HFUBC [42.336%], US [24.174%], FUBC18-IDV & HFUBC [29.858%], NEIGHBOR [1.125%]
maizefor	288033	MFM [17.299%], FUBC18-AGG [25.223%], HFUBC [41.525%], GB [1.733%], FUBC18-IDV & HFUBC [14.145%], NEIGHBOR [0.075%]
mango	612851	MFM [98.771%], FUBC18-AGG [1.082%], NEIGHBOR [0.146%]
mate	10613	CROPGROUP [100%]
melonetc	136761	MFM [95.948%], FUBC18-IDV [0.331%], NEIGHBOR [3.721%]
melonseed	13206	CROPGROUP [100%]
millet	234711	MFM [80.984%], FUBC18-IDV [17.925%], FUBC18-AGG [0.601%], NEIGHBOR [0.489%]
mixedgrain	45568	MFM [99.666%], NEIGHBOR [0.334%]
mixedgrass	249210	MFM [99.971%], NEIGHBOR [0.029%]
mushroom	33404	MFM [98.330%], NEIGHBOR [1.670%]
mustard	11957	MFM [99.632%], NEIGHBOR [0.368%]
nutmeg	29962	CROPGROUP [100%]
nutnes	8406	MFM [99.921%], NEIGHBOR [0.079%]
oats	390989	MFM [57.493%], FUBC18-AGG [38.670%], GB [3.719%], NEIGHBOR [0.118%]
oilpalm	2114949	MFM [6.467%], HFUBC [0.914%], FUBC18-IDV & HFUBC [91.590%], NEIGHBOR [1.030%]
oilseedfor	10367	MFM [87.640%], NEIGHBOR [12.360%]
oilseednes	37142	MFM [97.028%], NEIGHBOR [2.972%]
okra	47668	MFM [98.566%], NEIGHBOR [1.434%]
olive	479470	MFM [98.635%], FUBC18-IDV [0.463%], NEIGHBOR [0.903%]
onion	523357	MFM [99.261%], FUBC18-IDV [0.242%], FUBC18-AGG [0.063%], NEIGHBOR [0.433%]
orange	527627	MFM [67.113%], FUBC18-IDV [11.896%], FUBC18-AGG [20.809%], NEIGHBOR [0.182%]
papaya	21328	MFM [99.929%], NEIGHBOR [0.071%]
pea	214886	MFM [98.542%], NEIGHBOR [1.458%]
peachetc	241723	MFM [99.784%], NEIGHBOR [0.216%]
pear	164943	MFM [99.945%], NEIGHBOR [0.055%]

pepper	44612	MFM [99.831%], FUBC18-AGG [0.160%], NEIGHBOR [0.009%]
peppermint	2051	CROPGROUP [100%]
persimmon	230974	MFM [99.818%], NEIGHBOR [0.182%]
pigeonpea	48370	MFM [99.961%], NEIGHBOR [0.039%]
pimento	101517	CROPGROUP [100%]
pineapple	70390	MFM [87.273%], FUBC18-IDV [9.233%], FUBC18-AGG [3.090%], NEIGHBOR [0.405%]
pistachio	88120	MFM [99.936%], NEIGHBOR [0.064%]
plantain	33599	MFM [99.433%], NEIGHBOR [0.567%]
plum	113434	MFM [99.974%], NEIGHBOR [0.026%]
popcorn	10170	CROPGROUP [100%]
poppy	3325	MFM [99.987%], NEIGHBOR [0.013%]
potato	1639897	MFM [44.567%], FUBC18-IDV [14.086%], FUBC18-AGG [39.280%], BY [0.218%], GB [1.017%], NEIGHBOR [0.832%]
pulsenes	124966	MFM [98.594%], NEIGHBOR [1.406%]
pumpkinetc	203722	MFM [99.423%], NEIGHBOR [0.577%]
pyrethrum	187	CROPGROUP [100%]
quince	2823	MFM [99.834%], NEIGHBOR [0.166%]
quinoa	7266	CROPGROUP [100%]
ramie	9912	CROPGROUP [100%]
rapeseed	3321221	MFM [1.743%], FUBC18-AGG [3.585%], HFUBC [21.494%], GB [1.496%], FUBC18-IDV & HFUBC [70.854%], NEIGHBOR [0.829%]
rasberry	7694	MFM [99.304%], NEIGHBOR [0.696%]
rice	17448180	MFM [3.199%], FUBC18-AGG [0.192%], HFUBC [1.216%], AU [0.009%], FUBC18-IDV & HFUBC [95.230%], NEIGHBOR [0.155%]
rootnes	61553	MFM [99.139%], NEIGHBOR [0.861%]
rubber	314870	MFM [66.013%], FUBC18-IDV [33.958%], NEIGHBOR [0.029%]
rye	227050	MFM [35.421%], FUBC18-AGG [63.494%], GB [1.022%], NEIGHBOR [0.063%]
ryefor	37619	MFM [30.063%], NEIGHBOR [69.937%]
safflower	24040	MFM [99.988%], NEIGHBOR [0.012%]
sesame	351822	MFM [98.732%], FUBC18-IDV [0.016%], NEIGHBOR [1.252%]
sisal	360	MFM [99.982%], NEIGHBOR [0.018%]
sorghum	605058	MFM [84.480%], FUBC18-IDV [14.875%], FUBC18-AGG [0.195%], NEIGHBOR [0.450%]
sorghumfor	23213	MFM [99.248%], NEIGHBOR [0.752%]
sourcherry	10634	MFM [99.581%], NEIGHBOR [0.419%]
soybean	1936109	MFM [0.852%], FUBC18-IDV [0.801%], FUBC18-AGG [2.192%], HFUBC [1.185%], US [8.914%], FUBC18-IDV & HFUBC [85.904%], NEIGHBOR [0.152%]
spicenes	71625	CROPGROUP [100%]
spinach	148575	MFM [99.802%], FUBC18-AGG [0.000%], NEIGHBOR [0.198%]
stonefruitnes	6525	MFM [99.065%], NEIGHBOR [0.935%]
strawberry	23854	MFM [99.974%], NEIGHBOR [0.026%]
stringbean	7689	MFM [97.495%], NEIGHBOR [2.505%]
sugarbeet	589763	MFM [1.977%], FUBC18-AGG [10.490%], HFUBC [31.278%], BY [2.072%], GB [1.301%], FUBC18-IDV & HFUBC [52.501%], NEIGHBOR [0.381%]
sugarcane	3174971	MFM [3.604%], FUBC18-AGG [4.302%], HFUBC [0.244%], AU [3.617%], FUBC18-IDV & HFUBC [87.998%], NEIGHBOR [0.235%]
sugarnes	9945	CROPGROUP [100%]
sunflower	863066	MFM [35.976%], FUBC18-IDV [33.951%], FUBC18-AGG [29.969%], NEIGHBOR [0.104%]
swedefor	4090	CROPGROUP [100%]
sweetpotato	428960	MFM [18.807%], FUBC18-AGG [81.018%], NEIGHBOR [0.175%]
tangetc	477952	MFM [99.757%], FUBC18-AGG [0.098%], NEIGHBOR [0.145%]
taro	26874	MFM [91.624%], FUBC18-IDV [8.375%], NEIGHBOR [0.001%]
tea	1169193	MFM [7.752%], HFUBC [0.091%], FUBC18-IDV & HFUBC [92.137%], NEIGHBOR [0.020%]
tobacco	301744	MFM [70.751%], FUBC18-IDV [28.972%], NEIGHBOR [0.277%]
tomato	521518	MFM [99.898%], FUBC18-IDV [0.074%], FUBC18-AGG [0.002%], NEIGHBOR [0.026%]
triticale	202284	MFM [25.123%], FUBC18-AGG [64.507%], GB [0.499%], NEIGHBOR [9.871%]
tropicalnes	132535	MFM [99.616%], NEIGHBOR [0.384%]
tung	8662	CROPGROUP [100%]
turnipfor	7039	MFM [98.685%], NEIGHBOR [1.315%]
vanilla	1076	CROPGROUP [100%]

vegetablenes	767828	MFM [99.566%], NEIGHBOR [0.434%]
vegfor	889976	MFM [10.542%], NEIGHBOR [89.458%]
vetch	8315	CROPGROUP [100%]
walnut	2285	MFM [99.877%], NEIGHBOR [0.123%]
watermelon	399741	MFM [99.458%], NEIGHBOR [0.542%]
wheat	19004357	MFM [3.842%], HFUBC [15.877%], US [5.655%], GB [1.152%], FUBC18-IDV & HFUBC [72.332%], NEIGHBOR [1.141%]
yam	23374	MFM [17.695%], FUBC18-IDV [76.921%], NEIGHBOR [5.384%]
yautia	885	CROPGROUP [100%]

Supplementary Table 6 The selected best-fit datasets used to construct the global maps of P_2O_5 application rates of each individual crop.

Crops	Applied Mass [tonnes]	Datasets selected [% contribution of the dataset to the global map]
abaca	752	CROPGROUP [100%]
agave	934	CROPGROUP [100%]
alfalfa	460114	MFM [98.702%], FUBC18-IDV [0.900%], NEIGHBOR [0.398%]
almond	38589	MFM [99.717%], NEIGHBOR [0.283%]
aniseetc	62594	CROPGROUP [100%]
apple	282439	MFM [23.210%], FUBC18-AGG [76.344%], NEIGHBOR [0.445%]
apricot	9330	MFM [99.684%], NEIGHBOR [0.316%]
areca	11257	CROPGROUP [100%]
artichoke	7200	MFM [94.580%], NEIGHBOR [5.420%]
asparagus	102929	MFM [99.949%], NEIGHBOR [0.051%]
avocado	26398	MFM [99.999%], NEIGHBOR [0.001%]
bambara	330	CROPGROUP [100%]
banana	122318	MFM [96.186%], FUBC18-IDV [0.802%], NEIGHBOR [3.011%]
barley	864662	MFM [65.598%], FUBC18-IDV [29.679%], GB [4.227%], NEIGHBOR [0.495%]
bean	581027	MFM [99.767%], FUBC18-IDV [0.087%], NEIGHBOR [0.145%]
beetfor	6835	MFM [99.404%], NEIGHBOR [0.596%]
berrynes	2382	MFM [98.696%], NEIGHBOR [1.304%]
blueberry	2137	MFM [89.125%], NEIGHBOR [10.875%]
brazil	104	CROPGROUP [100%]
broadbean	91497	MFM [98.546%], NEIGHBOR [1.454%]
buckwheat	41865	MFM [99.795%], NEIGHBOR [0.205%]
cabbage	118484	MFM [99.665%], FUBC18-AGG [0.007%], NEIGHBOR [0.328%]
cabbagefor	5300	MFM [99.994%], NEIGHBOR [0.006%]
canaryseed	4674	MFM [99.814%], NEIGHBOR [0.186%]
carob	3250	MFM [99.980%], NEIGHBOR [0.020%]
carrot	55384	MFM [98.058%], FUBC18-AGG [0.000%], NEIGHBOR [1.942%]
carrotfor	20	CROPGROUP [100%]
cashew	55569	CROPGROUP [100%]
cashewapple	8526	MFM [99.981%], NEIGHBOR [0.019%]
cassava	145641	MFM [72.477%], FUBC18-IDV [26.756%], NEIGHBOR [0.766%]
castor	15405	MFM [99.950%], NEIGHBOR [0.050%]
cauliflower	70065	MFM [99.165%], NEIGHBOR [0.835%]
cerealnes	29210	MFM [96.335%], NEIGHBOR [3.665%]
cherry	8561	MFM [99.992%], NEIGHBOR [0.008%]
chestnut	12917	MFM [99.944%], NEIGHBOR [0.056%]
chickpea	72430	MFM [98.833%], NEIGHBOR [1.167%]
chicory	770	CROPGROUP [100%]
chilleetc	73429	CROPGROUP [100%]
cinnamon	11402	CROPGROUP [100%]
citrusnes	27497	MFM [98.241%], NEIGHBOR [1.759%]
clove	19358	CROPGROUP [100%]
clover	18283	MFM [83.227%], NEIGHBOR [16.773%]
cocoa	85234	MFM [92.698%], FUBC18-IDV [6.982%], NEIGHBOR [0.320%]
coconut	28522	MFM [70.655%], FUBC18-IDV [26.819%], NEIGHBOR [2.526%]
coffee	259174	MFM [49.800%], FUBC18-IDV [0.000%], HFUBC [0.110%], FUBC18-IDV & HFUBC [50.003%], NEIGHBOR [0.088%]
cotton	1618591	MFM [3.095%], FUBC18-IDV [8.033%], HFUBC [2.664%], US [6.277%], FUBC18-IDV & HFUBC [79.771%], NEIGHBOR [0.161%]
cowpea	14462	MFM [69.222%], FUBC18-IDV [16.198%], NEIGHBOR [14.579%]
cranberry	1148	MFM [99.479%], NEIGHBOR [0.521%]
cucumberetc	110464	MFM [99.624%], NEIGHBOR [0.376%]
currant	2091	MFM [99.990%], NEIGHBOR [0.010%]
date	56293	MFM [99.618%], NEIGHBOR [0.382%]
eggplant	88243	MFM [99.159%], NEIGHBOR [0.841%]
fibrenes	2699	CROPGROUP [100%]
fig	4807	MFM [99.023%], NEIGHBOR [0.977%]
flax	9055	MFM [80.093%], BY [12.649%], NEIGHBOR [7.258%]
fonio	606	FUBC18-IDV [0.873%], NEIGHBOR [99.127%]

fornes	199155	MFM [99.154%], NEIGHBOR [0.846%]
fruitnes	95351	MFM [99.734%], NEIGHBOR [0.266%]
garlic	81665	MFM [97.249%], FUBC18-IDV [0.305%], FUBC18-AGG [1.388%], NEIGHBOR [1.058%]
ginger	1972	FUBC18-IDV [22.491%], NEIGHBOR [77.509%]
gooseberry	506	MFM [99.970%], NEIGHBOR [0.030%]
grape	204359	MFM [71.051%], FUBC18-IDV [1.982%], FUBC18-AGG [26.847%], NEIGHBOR [0.120%]
grapefruitetc	17454	MFM [99.954%], NEIGHBOR [0.046%]
grassnes	70633	MFM [98.560%], NEIGHBOR [1.440%]
greenbean	70974	MFM [98.614%], NEIGHBOR [1.386%]
greenbroadbean	7018	MFM [83.243%], NEIGHBOR [16.757%]
greencorn	59572	MFM [71.613%], FUBC18-IDV [2.824%], HFUBC [6.009%], NEIGHBOR [19.554%]
greenonion	11710	MFM [95.574%], NEIGHBOR [4.426%]
greenpea	130769	MFM [97.757%], GB [0.316%], NEIGHBOR [1.927%]
groundnut	605400	MFM [97.255%], FUBC18-IDV [2.683%], NEIGHBOR [0.063%]
hazelnut	17425	MFM [99.844%], NEIGHBOR [0.156%]
hemp	1719	MFM [90.376%], NEIGHBOR [9.624%]
hempseed	1203	CROPGROUP [100%]
hop	2435	MFM [98.402%], NEIGHBOR [1.598%]
jute	27516	MFM [51.776%], FUBC18-IDV [48.143%], NEIGHBOR [0.080%]
jutelikefiber	8625	MFM [99.882%], NEIGHBOR [0.118%]
kapokfiber	16653	CROPGROUP [100%]
kapokseed	8176	CROPGROUP [100%]
karite	902	CROPGROUP [100%]
kiwi	3998	MFM [99.121%], NEIGHBOR [0.879%]
kolanut	899	CROPGROUP [100%]
legumenes	14500	MFM [99.874%], NEIGHBOR [0.126%]
lemonlime	38258	MFM [98.160%], FUBC18-AGG [1.584%], NEIGHBOR [0.256%]
lentil	118515	MFM [99.855%], NEIGHBOR [0.145%]
lettuce	75884	MFM [99.777%], NEIGHBOR [0.223%]
linseed	30530	MFM [93.457%], FUBC18-AGG [4.610%], GB [0.367%], NEIGHBOR [1.565%]
lupin	10535	MFM [99.411%], NEIGHBOR [0.589%]
maize	9168051	MFM [1.984%], HFUBC [51.250%], US [20.394%], FUBC18-IDV & HFUBC [25.676%], NEIGHBOR [0.696%]
maizefor	94099	MFM [15.783%], FUBC18-AGG [26.617%], HFUBC [32.924%], GB [2.995%], FUBC18-IDV & HFUBC [21.579%], NEIGHBOR [0.103%]
mango	139702	MFM [94.324%], FUBC18-AGG [5.525%], NEIGHBOR [0.151%]
mate	3183	CROPGROUP [100%]
melonetc	62823	MFM [96.409%], FUBC18-IDV [0.288%], NEIGHBOR [3.303%]
melonseed	4360	CROPGROUP [100%]
millet	72412	MFM [87.430%], FUBC18-IDV [9.939%], FUBC18-AGG [1.947%], NEIGHBOR [0.683%]
mixedgrain	15288	MFM [99.634%], NEIGHBOR [0.366%]
mixedgrass	155129	MFM [99.982%], NEIGHBOR [0.018%]
mushroom	13228	MFM [97.914%], NEIGHBOR [2.086%]
mustard	2439	MFM [97.705%], NEIGHBOR [2.295%]
nutmeg	11523	CROPGROUP [100%]
nutnes	13626	MFM [99.874%], NEIGHBOR [0.126%]
oats	152449	MFM [71.945%], FUBC18-AGG [25.166%], GB [2.806%], NEIGHBOR [0.083%]
oilpalm	1007550	MFM [3.842%], HFUBC [0.722%], FUBC18-IDV & HFUBC [94.284%], NEIGHBOR [1.151%]
oilseedfor	9069	MFM [99.969%], NEIGHBOR [0.031%]
oilseednes	25426	MFM [97.488%], NEIGHBOR [2.512%]
okra	31127	MFM [98.627%], NEIGHBOR [1.373%]
olive	181138	MFM [98.362%], FUBC18-IDV [0.328%], NEIGHBOR [1.310%]
onion	196964	MFM [97.524%], FUBC18-IDV [0.630%], FUBC18-AGG [0.083%], NEIGHBOR [1.764%]
orange	157611	MFM [58.956%], FUBC18-IDV [15.425%], FUBC18-AGG [25.416%], NEIGHBOR [0.203%]
papaya	7464	MFM [99.938%], NEIGHBOR [0.062%]
pea	184506	MFM [98.712%], GB [0.399%], NEIGHBOR [0.889%]
peachetc	62566	MFM [99.774%], NEIGHBOR [0.226%]
pear	80347	MFM [99.956%], NEIGHBOR [0.044%]

pepper	17675	MFM [99.780%], FUBC18-AGG [0.199%], NEIGHBOR [0.020%]
peppermint	1021	CROPGROUP [100%]
persimmon	89461	MFM [99.836%], NEIGHBOR [0.164%]
pigeonpea	40207	MFM [99.962%], NEIGHBOR [0.038%]
pimento	29357	CROPGROUP [100%]
pineapple	32907	MFM [88.810%], FUBC18-IDV [3.037%], FUBC18-AGG [7.737%], NEIGHBOR [0.417%]
pistachio	58901	MFM [99.937%], NEIGHBOR [0.063%]
plantain	13112	MFM [99.256%], NEIGHBOR [0.744%]
plum	44386	MFM [99.986%], NEIGHBOR [0.014%]
popcorn	3635	CROPGROUP [100%]
poppy	1434	MFM [99.976%], NEIGHBOR [0.024%]
potato	787622	MFM [35.714%], FUBC18-IDV [12.552%], FUBC18-AGG [48.655%], BY [0.755%], GB [1.612%], NEIGHBOR [0.713%]
pulsenes	125733	MFM [98.206%], NEIGHBOR [1.794%]
pumpkinetc	79316	MFM [98.266%], NEIGHBOR [1.734%]
pyrethrum	94	CROPGROUP [100%]
quince	1306	MFM [99.799%], NEIGHBOR [0.201%]
quinoa	3317	CROPGROUP [100%]
ramie	3592	CROPGROUP [100%]
rapeseed	1281782	MFM [1.337%], FUBC18-AGG [4.246%], HFUBC [12.713%], GB [0.696%], FUBC18-IDV & HFUBC [79.940%], NEIGHBOR [1.068%]
rasberry	1947	MFM [98.826%], NEIGHBOR [1.174%]
rice	6854775	MFM [2.379%], FUBC18-AGG [0.144%], HFUBC [1.311%], FUBC18-IDV & HFUBC [96.034%], NEIGHBOR [0.131%]
rootnes	44063	MFM [98.710%], NEIGHBOR [1.290%]
rubber	130844	MFM [91.151%], FUBC18-IDV [8.829%], NEIGHBOR [0.020%]
rye	52855	MFM [41.309%], FUBC18-AGG [58.232%], GB [0.412%], NEIGHBOR [0.048%]
ryefor	28022	MFM [63.592%], NEIGHBOR [36.408%]
safflower	6832	MFM [99.981%], NEIGHBOR [0.019%]
sesame	106506	MFM [98.048%], FUBC18-IDV [0.176%], NEIGHBOR [1.776%]
sisal	9496	MFM [99.990%], NEIGHBOR [0.010%]
sorghum	202598	MFM [87.893%], FUBC18-IDV [11.012%], FUBC18-AGG [0.583%], NEIGHBOR [0.512%]
sorghumfor	15990	MFM [99.932%], NEIGHBOR [0.068%]
sourcherry	2990	MFM [99.280%], NEIGHBOR [0.720%]
soybean	5818385	MFM [0.256%], FUBC18-AGG [0.293%], HFUBC [0.193%], US [14.177%], FUBC18-IDV & HFUBC [85.016%], NEIGHBOR [0.065%]
spicenes	20990	CROPGROUP [100%]
spinach	60987	MFM [99.828%], FUBC18-AGG [0.000%], NEIGHBOR [0.172%]
stonefruitnes	2032	MFM [99.390%], NEIGHBOR [0.610%]
strawberry	11062	MFM [99.961%], NEIGHBOR [0.039%]
stringbean	3933	MFM [97.553%], NEIGHBOR [2.447%]
sugarbeet	273489	MFM [1.296%], FUBC18-AGG [5.109%], HFUBC [27.368%], BY [3.443%], GB [0.895%], FUBC18-IDV & HFUBC [61.241%], NEIGHBOR [0.648%]
sugarcane	1164859	MFM [5.022%], FUBC18-AGG [4.429%], HFUBC [0.556%], FUBC18-IDV & HFUBC [89.688%], NEIGHBOR [0.305%]
sugarnes	3495	CROPGROUP [100%]
sunflower	317105	MFM [33.511%], FUBC18-IDV [38.822%], FUBC18-AGG [27.541%], NEIGHBOR [0.126%]
swedefor	2768	CROPGROUP [100%]
sweetpotato	238462	MFM [13.551%], FUBC18-AGG [86.209%], NEIGHBOR [0.240%]
tangetc	201402	MFM [99.592%], FUBC18-AGG [0.269%], NEIGHBOR [0.139%]
taro	11369	MFM [94.580%], FUBC18-IDV [5.414%], NEIGHBOR [0.007%]
tea	369775	MFM [5.309%], HFUBC [0.174%], FUBC18-IDV & HFUBC [94.502%], NEIGHBOR [0.015%]
tobacco	157969	MFM [73.600%], FUBC18-IDV [26.014%], NEIGHBOR [0.386%]
tomato	198862	MFM [98.937%], FUBC18-IDV [0.293%], FUBC18-AGG [0.024%], NEIGHBOR [0.746%]
triticale	60427	MFM [30.295%], FUBC18-AGG [54.287%], GB [0.157%], NEIGHBOR [15.261%]
tropicalnes	45868	MFM [99.504%], NEIGHBOR [0.496%]
tung	3447	CROPGROUP [100%]
turnipfor	5977	MFM [99.454%], NEIGHBOR [0.546%]
vanilla	413	CROPGROUP [100%]

vegetablenes	311739	MFM [99.123%], NEIGHBOR [0.877%]
vegfor	377808	MFM [11.381%], NEIGHBOR [88.619%]
vetch	10137	CROPGROUP [100%]
walnut	13793	MFM [99.407%], NEIGHBOR [0.593%]
watermelon	171058	MFM [99.689%], NEIGHBOR [0.311%]
wheat	6957198	MFM [5.421%], HFUBC [8.525%], US [4.946%], GB [0.366%], FUBC18-IDV & HFUBC [80.154%], NEIGHBOR [0.587%]
yam	7083	MFM [27.376%], FUBC18-IDV [67.301%], NEIGHBOR [5.324%]
yautia	614	CROPGROUP [100%]

Supplementary Table 7 The selected best-fit datasets used to construct the global maps of K_2O application rates of each individual crop.

Crops	Applied Mass [tonnes]	Datasets selected [% contribution of the dataset to the global map]
abaca	1662	CROPGROUP [100%]
agave	964	CROPGROUP [100%]
alfalfa	456341	MFM [99.121%], FUBC18-IDV [0.265%], NEIGHBOR [0.614%]
almond	37912	MFM [99.735%], NEIGHBOR [0.265%]
aniseetc	33195	CROPGROUP [100%]
apple	431306	MFM [15.008%], FUBC18-AGG [84.630%], NEIGHBOR [0.362%]
apricot	9083	MFM [99.684%], NEIGHBOR [0.316%]
areca	24202	CROPGROUP [100%]
artichoke	7202	MFM [94.420%], NEIGHBOR [5.580%]
asparagus	90857	MFM [99.942%], NEIGHBOR [0.058%]
avocado	25676	MFM [99.999%], NEIGHBOR [0.001%]
bambara	304	CROPGROUP [100%]
banana	123701	MFM [92.880%], FUBC18-IDV [4.409%], HFUBC [0.007%], NEIGHBOR [2.704%]
barley	871328	MFM [64.776%], FUBC18-IDV [29.073%], GB [5.718%], NEIGHBOR [0.433%]
bean	570720	MFM [99.853%], NEIGHBOR [0.147%]
beetfor	5653	MFM [99.284%], NEIGHBOR [0.716%]
berrynes	2331	MFM [98.708%], NEIGHBOR [1.292%]
blueberry	2087	MFM [88.866%], NEIGHBOR [11.134%]
brazil	104	CROPGROUP [100%]
broadbean	85216	MFM [98.472%], NEIGHBOR [1.528%]
buckwheat	36534	MFM [99.766%], NEIGHBOR [0.234%]
cabbage	111205	MFM [99.629%], FUBC18-AGG [0.009%], HFUBC [0.007%], NEIGHBOR [0.355%]
cabbagefor	4745	MFM [99.993%], NEIGHBOR [0.007%]
canaryseed	4661	MFM [99.819%], NEIGHBOR [0.181%]
carob	3195	MFM [99.980%], NEIGHBOR [0.020%]
carrot	53390	MFM [97.948%], FUBC18-AGG [0.000%], NEIGHBOR [2.052%]
carrotfor	19	CROPGROUP [100%]
cashew	54930	CROPGROUP [100%]
cashewapple	8061	MFM [99.976%], NEIGHBOR [0.024%]
cassava	183817	MFM [54.373%], FUBC18-IDV [44.202%], NEIGHBOR [1.425%]
castor	15074	MFM [99.949%], NEIGHBOR [0.051%]
cauliflower	67072	MFM [99.098%], NEIGHBOR [0.902%]
cerealnes	29543	MFM [96.376%], NEIGHBOR [3.624%]
cherry	8505	MFM [99.992%], NEIGHBOR [0.008%]
chestnut	11771	MFM [99.941%], NEIGHBOR [0.059%]
chickpea	71631	MFM [98.828%], NEIGHBOR [1.172%]
chicory	819	CROPGROUP [100%]
chilleetc	69265	CROPGROUP [100%]
cinnamon	16977	CROPGROUP [100%]
citrusnes	26104	MFM [98.149%], NEIGHBOR [1.851%]
clove	49511	CROPGROUP [100%]
clover	17397	MFM [82.582%], NEIGHBOR [17.418%]
cocoa	83206	MFM [94.349%], FUBC18-IDV [5.323%], NEIGHBOR [0.328%]
coconut	35115	MFM [57.189%], FUBC18-IDV [40.765%], HFUBC [0.025%], NEIGHBOR [2.021%]
coffee	405692	MFM [31.617%], HFUBC [0.144%], FUBC18-IDV & HFUBC [68.206%], NEIGHBOR [0.033%]
cotton	898320	MFM [5.533%], HFUBC [4.719%], US [12.682%], FUBC18-IDV & HFUBC [76.910%], NEIGHBOR [0.157%]
cowpea	13593	MFM [73.515%], FUBC18-IDV [10.998%], NEIGHBOR [15.487%]
cranberry	1212	MFM [99.507%], NEIGHBOR [0.493%]
cucumberetc	99890	MFM [99.577%], NEIGHBOR [0.423%]
currant	2045	MFM [99.991%], NEIGHBOR [0.009%]
date	52344	MFM [99.609%], NEIGHBOR [0.391%]
eggplant	81153	MFM [99.074%], NEIGHBOR [0.926%]
fibrenes	2246	CROPGROUP [100%]
fig	4735	MFM [99.008%], NEIGHBOR [0.992%]
flax	8914	MFM [80.157%], BY [12.471%], NEIGHBOR [7.372%]
fonio	606	FUBC18-IDV [0.863%], NEIGHBOR [99.137%]

fornes	192887	MFM [99.132%], NEIGHBOR [0.868%]
fruitnes	90442	MFM [99.725%], NEIGHBOR [0.275%]
garlic	74039	MFM [96.875%], FUBC18-IDV [0.224%], FUBC18-AGG [1.696%], NEIGHBOR [1.205%]
ginger	1699	FUBC18-IDV [22.915%], NEIGHBOR [77.085%]
gooseberry	505	MFM [99.970%], NEIGHBOR [0.030%]
grape	192483	MFM [68.497%], FUBC18-IDV [1.373%], FUBC18-AGG [30.002%], NEIGHBOR [0.128%]
grapefruitetc	16553	MFM [99.955%], NEIGHBOR [0.045%]
grassnes	66909	MFM [98.493%], NEIGHBOR [1.507%]
greenbean	66495	MFM [98.476%], NEIGHBOR [1.524%]
greenbroadbean	7004	MFM [82.474%], NEIGHBOR [17.526%]
greencorn	64469	MFM [72.049%], FUBC18-IDV [2.603%], HFUBC [6.872%], NEIGHBOR [18.476%]
greenonion	13726	MFM [95.525%], NEIGHBOR [4.475%]
greenpea	120832	MFM [97.490%], GB [0.342%], NEIGHBOR [2.168%]
groundnut	537140	MFM [97.533%], FUBC18-IDV [2.397%], NEIGHBOR [0.070%]
hazelnut	17205	MFM [99.843%], NEIGHBOR [0.157%]
hemp	1672	MFM [90.110%], NEIGHBOR [9.890%]
hempseed	789	CROPGROUP [100%]
hop	2377	MFM [98.997%], NEIGHBOR [1.003%]
jute	27273	MFM [50.517%], FUBC18-IDV [49.404%], NEIGHBOR [0.080%]
jutelikefiber	8257	MFM [99.877%], NEIGHBOR [0.123%]
kapokfiber	42876	CROPGROUP [100%]
kapokseed	21527	CROPGROUP [100%]
karite	902	CROPGROUP [100%]
kiwi	3778	MFM [99.259%], NEIGHBOR [0.741%]
kolanut	899	CROPGROUP [100%]
legumenes	14326	MFM [99.874%], NEIGHBOR [0.126%]
lemonlime	36894	MFM [97.350%], FUBC18-AGG [2.386%], NEIGHBOR [0.264%]
lentil	117845	MFM [99.855%], NEIGHBOR [0.145%]
lettuce	72653	MFM [99.758%], NEIGHBOR [0.242%]
linseed	29327	MFM [92.290%], FUBC18-AGG [6.161%], NEIGHBOR [1.549%]
lupin	10513	MFM [99.416%], NEIGHBOR [0.584%]
maize	6831600	MFM [4.651%], HFUBC [45.656%], US [27.669%], FUBC18-IDV & HFUBC [21.399%], NEIGHBOR [0.625%]
maizefor	102646	MFM [13.783%], FUBC18-AGG [26.160%], HFUBC [32.558%], GB [1.882%], FUBC18-IDV & HFUBC [25.516%], NEIGHBOR [0.101%]
mango	140362	MFM [91.862%], FUBC18-AGG [7.988%], NEIGHBOR [0.149%]
mate	7494	CROPGROUP [100%]
melonetc	59772	MFM [96.012%], FUBC18-IDV [0.379%], NEIGHBOR [3.610%]
melonseed	3790	CROPGROUP [100%]
millet	69843	MFM [87.364%], FUBC18-IDV [9.915%], FUBC18-AGG [2.019%], NEIGHBOR [0.702%]
mixedgrain	15236	MFM [99.633%], NEIGHBOR [0.367%]
mixedgrass	151529	MFM [99.982%], NEIGHBOR [0.018%]
mushroom	12121	MFM [97.407%], NEIGHBOR [2.593%]
mustard	2384	MFM [97.669%], NEIGHBOR [2.331%]
nutmeg	24194	CROPGROUP [100%]
nutnes	13446	MFM [99.875%], NEIGHBOR [0.125%]
oats	155043	MFM [70.259%], FUBC18-AGG [26.256%], GB [3.409%], NEIGHBOR [0.076%]
oilpalm	3443272	MFM [1.114%], HFUBC [0.811%], FUBC18-IDV & HFUBC [97.166%], NEIGHBOR [0.909%]
oilseedfor	9064	MFM [99.969%], NEIGHBOR [0.031%]
oilseednes	26868	MFM [97.872%], NEIGHBOR [2.128%]
okra	31618	MFM [98.717%], NEIGHBOR [1.283%]
olive	178307	MFM [97.939%], FUBC18-IDV [0.742%], NEIGHBOR [1.319%]
onion	190984	MFM [97.621%], FUBC18-IDV [0.433%], FUBC18-AGG [0.095%], NEIGHBOR [1.851%]
orange	197259	MFM [47.742%], FUBC18-IDV [13.469%], FUBC18-AGG [38.599%], NEIGHBOR [0.190%]
papaya	7513	MFM [99.939%], NEIGHBOR [0.061%]
pea	178022	MFM [98.800%], GB [0.268%], NEIGHBOR [0.933%]
peachetc	56316	MFM [99.750%], NEIGHBOR [0.250%]
pear	70872	MFM [99.953%], NEIGHBOR [0.047%]

pepper	17635	MFM [99.758%], FUBC18-AGG [0.221%], NEIGHBOR [0.020%]
peppermint	1056	CROPGROUP [100%]
persimmon	86463	MFM [99.768%], NEIGHBOR [0.232%]
pigeonpea	39692	MFM [99.962%], NEIGHBOR [0.038%]
pimento	29807	CROPGROUP [100%]
pineapple	39745	MFM [76.693%], FUBC18-IDV [13.676%], FUBC18-AGG [9.293%], NEIGHBOR [0.339%]
pistachio	58776	MFM [99.942%], NEIGHBOR [0.058%]
plantain	13058	MFM [99.259%], NEIGHBOR [0.741%]
plum	39245	MFM [99.985%], NEIGHBOR [0.015%]
popcorn	3283	CROPGROUP [100%]
poppy	1374	MFM [99.976%], NEIGHBOR [0.024%]
potato	850407	MFM [36.517%], FUBC18-IDV [20.262%], FUBC18-AGG [38.912%], BY [1.082%], GB [2.521%], NEIGHBOR [0.706%]
pulsenes	124556	MFM [98.199%], NEIGHBOR [1.801%]
pumpkinetc	79406	MFM [98.213%], NEIGHBOR [1.787%]
pyrethrum	97	CROPGROUP [100%]
quince	1214	MFM [99.784%], NEIGHBOR [0.216%]
quinoa	1866	CROPGROUP [100%]
ramie	1788	CROPGROUP [100%]
rapeseed	801112	MFM [2.244%], FUBC18-AGG [2.064%], HFUBC [27.379%], GB [0.932%], FUBC18-IDV & HFUBC [65.505%], NEIGHBOR [1.875%]
rasberry	1928	MFM [98.819%], NEIGHBOR [1.181%]
rice	4803336	MFM [3.525%], FUBC18-AGG [0.216%], HFUBC [1.862%], FUBC18-IDV & HFUBC [94.357%], NEIGHBOR [0.040%]
rootnes	49622	MFM [98.486%], NEIGHBOR [1.514%]
rubber	147322	MFM [80.428%], FUBC18-IDV [19.555%], NEIGHBOR [0.017%]
rye	59136	MFM [35.311%], FUBC18-AGG [64.159%], GB [0.491%], NEIGHBOR [0.040%]
ryefor	27944	MFM [63.621%], NEIGHBOR [36.379%]
safflower	6656	MFM [99.981%], NEIGHBOR [0.019%]
sesame	103112	MFM [98.146%], FUBC18-IDV [0.090%], NEIGHBOR [1.764%]
sisal	9303	MFM [99.990%], NEIGHBOR [0.010%]
sorghum	195506	MFM [87.925%], FUBC18-IDV [10.945%], FUBC18-AGG [0.604%], NEIGHBOR [0.527%]
sorghumfor	14541	MFM [99.928%], NEIGHBOR [0.072%]
sourcherry	2975	MFM [99.277%], NEIGHBOR [0.723%]
soybean	5543563	MFM [1.295%], FUBC18-AGG [0.216%], HFUBC [0.290%], US [24.161%], FUBC18-IDV & HFUBC [73.982%], NEIGHBOR [0.057%]
spicenes	20951	CROPGROUP [100%]
spinach	56093	MFM [99.818%], FUBC18-AGG [0.000%], NEIGHBOR [0.182%]
stonefruitnes	1961	MFM [99.368%], NEIGHBOR [0.632%]
strawberry	11401	MFM [99.962%], NEIGHBOR [0.038%]
stringbean	3870	MFM [97.725%], NEIGHBOR [2.275%]
sugarbeet	297014	MFM [1.240%], FUBC18-AGG [3.139%], HFUBC [43.585%], BY [5.814%], GB [1.685%], FUBC18-IDV & HFUBC [43.927%], NEIGHBOR [0.610%]
sugarcane	1689911	MFM [3.543%], FUBC18-AGG [4.536%], HFUBC [0.896%], FUBC18-IDV & HFUBC [90.821%], NEIGHBOR [0.204%]
sugarnes	7984	CROPGROUP [100%]
sunflower	284023	MFM [35.939%], FUBC18-IDV [43.705%], FUBC18-AGG [20.139%], NEIGHBOR [0.217%]
swedefor	3323	CROPGROUP [100%]
sweetpotato	212451	MFM [15.895%], FUBC18-AGG [83.764%], NEIGHBOR [0.340%]
tangetc	187742	MFM [99.442%], FUBC18-AGG [0.420%], NEIGHBOR [0.138%]
taro	11372	MFM [94.839%], FUBC18-IDV [5.155%], NEIGHBOR [0.007%]
tea	424515	MFM [4.675%], HFUBC [0.280%], FUBC18-IDV & HFUBC [95.033%], NEIGHBOR [0.013%]
tobacco	155097	MFM [72.392%], FUBC18-IDV [26.924%], NEIGHBOR [0.684%]
tomato	199199	MFM [98.655%], FUBC18-IDV [0.534%], FUBC18-AGG [0.029%], NEIGHBOR [0.783%]
triticale	66594	MFM [26.483%], FUBC18-AGG [60.431%], GB [0.190%], NEIGHBOR [12.897%]
tropicalnes	42953	MFM [99.472%], NEIGHBOR [0.528%]
tung	3124	CROPGROUP [100%]
turnipfor	6993	MFM [99.547%], NEIGHBOR [0.453%]
vanilla	404	CROPGROUP [100%]

vegetablenes	305120	MFM [99.077%], NEIGHBOR [0.923%]
vegfor	350403	MFM [11.129%], NEIGHBOR [88.871%]
vetch	10100	CROPGROUP [100%]
walnut	13075	MFM [99.381%], NEIGHBOR [0.619%]
watermelon	161913	MFM [99.653%], NEIGHBOR [0.347%]
wheat	3451509	MFM [20.120%], HFUBC [12.727%], US [2.797%], GB [0.807%], FUBC18-IDV & HFUBC [63.426%], NEIGHBOR [0.123%]
yam	7598	MFM [35.709%], FUBC18-IDV [58.868%], NEIGHBOR [5.422%]
yautia	631	CROPGROUP [100%]
